# Nanoscale Tungsten-Microbial Interface of the Metal Immobilizing Thermoacidophilic Archaeon *Metallosphaera sedula* Cultivated With Tungsten Polyoxometalate

**DOI:** 10.3389/fmicb.2019.01267

**Published:** 2019-06-07

**Authors:** Tetyana Milojevic, Mihaela Albu, Amir Blazevic, Nadiia Gumerova, Lukas Konrad, Norbert Cyran

**Affiliations:** ^1^Extremophiles/Space Biochemistry Group, Department of Biophysical Chemistry, University of Vienna, Vienna, Austria; ^2^Graz Centre for Electron Microscopy, Graz, Austria; ^3^Department of Biophysical Chemistry, University of Vienna, Vienna, Austria; ^4^Core Facility Cell Imaging and Ultrastructure Research, University of Vienna, Vienna, Austria

**Keywords:** archaea, biomineralisation, *Metallosphaera sedula*, microbe–mineral interactions, tungsten

## Abstract

Inorganic systems based upon polyoxometalate (POM) clusters provide an experimental approach to develop artificial life. These artificial symmetric anionic macromolecules with oxidometalate polyhedra as building blocks were shown to be well suited as inorganic frameworks for complex self-assembling and organizing systems with emergent properties. Analogously to mineral cells based on iron sulfides, POMs are considered as inorganic cells in facilitating prelife chemical processes and displaying “life-like” characteristics. However, the relevance of POMs to life-sustaining processes (e.g., microbial respiration) has not yet been addressed, while iron sulfides are very well known as ubiquitous mineral precursors and energy sources for chemolithotrophic metabolism. *Metallosphaera sedula* is an extreme metallophilic and thermoacidophilic archaeon, which flourishes in hot acid and respires by metal oxidation. In the present study we provide our observations on *M. sedula* cultivated on tungsten polyoxometalate (W-POM). The decomposition of W-POM macromolecular clusters and the appearance of low molecular weight W species (e.g., WO) in the presence of *M. sedula* have been detected by electrospray ionization mass spectrometry (ESI-MS) analysis. Here, we document the presence of metalloorganic assemblages at the interface between *M. sedula* and W-POM resolved down to the nanometer scale using scanning and transmission electron microscopy (SEM and TEM) coupled to electron energy loss spectroscopy (EELS). High-resolution TEM (HR-TEM) and selected-area electron diffraction (SAED) patterns indicated the deposition of redox heterogeneous tungsten species on the S-layer of *M. sedula* along with the accumulation of intracellular tungsten-bearing nanoparticles, i.e., clusters of tungsten atoms. These results reveal the effectiveness of the analytical spectroscopy coupled to the wet chemistry approach as a tool in the analysis of metal–microbial interactions and microbial cultivation on supramolecular self-assemblages based on inorganic metal clusters. We discuss the possible mechanism of W-POM decomposition by *M. sedula* in light of unique electrochemical properties of POMs. The findings presented herein highlight unique metallophilicity in hostile environments, extending our knowledge of the relevance of POMs to life-sustaining processes, understanding of the transition of POMs as inorganic prebiotic model to life-sustainable material precursors and revealing biogenic signatures obtained after the decomposition of an artificial inorganic compound, which previously was not associated with any living matter.

## Introduction

Chemolithotrophy is the most ancient metabolic form of life, which enables the transition of energy from non-living inorganic mater to energy of living entity. Serving as a biochemical link between the mineral world and the last universal common ancestor, chemolithotrophic metabolism has permitted the evolving of prokaryotes into versatile energy scavengers. Chemolithotrophic microorganisms employ an astonishing number of metabolic pathways to extract energy from diverse inorganic electron donors and acceptors, which has significant consequences for global biogeochemical cycles. The extreme thermoacidophile *Metallosphaera sedula* is a metal mobilizing archaeon, which thrives in hot acid environments (optimal growth at 74°C and pH 2.0) and utilizes energy from the oxidation of metal inorganic sources (Huber et al., 1989; Peeples and Kelly, 1995; Auernik et al., 2008; Maezato et al., 2012; McCarthy et al., 2014; Reed, 2015; Wheaton et al., 2016; Kölbl et al., 2017). Apart from Fe-oxidizing properties, metabolically versatile *M. sedula* has the ability to use a variety of electron donors, including reduced inorganic sulfur compounds (Auernik and Kelly, 2008), uranium ores (Mukherjee et al., 2012), as well as molecular hydrogen (Auernik and Kelly, 2010). Chemolithoautotrophic growth of *M. sedula* has been shown on real and synthetic extraterrestrial multimetallic materials such as stony chondrite NWA 1172 (Reed, 2015) and Martian regolith simulants, where acquisition of Fe^2+^ and/or Mn^2+^ from these materials satisfy the bioenergetic needs of *M. sedula* (Kölbl et al., 2017).

Artificial polyoxometalates (POMs) are a family of anionic metal oxide clusters characterized by a variety of physical and chemical properties and functions in terms of molecular composition, size, shape, charge density and redox potential (Gumerova and Rompel, 2018). These artificial symmetric anionic macromolecules with oxidometalate polyhedra as building blocks were shown to be well suited as inorganic frameworks for complex self-assembling and -organizing systems with emergent properties (Long et al., 2007; Cogdell et al., 2010; Gao et al., 2011; Yin et al., 2012; Symes et al., 2013). Supramolecular self-assemblages based on polynuclear metal clusters of POMs have been proposed as micelle-like structures in developing approaches to artificial inorganic life. Analogously to mineral cells based on iron sulfide (Wächtershäuser, 1992), POMs are considered as inorganic mineral cells in facilitating prelife chemical processes and displaying “life-like” characteristics (Miras et al., 2010; Cooper et al., 2011). However, the relevance of POMs to life-sustaining processes (e.g., microbial respiration) has not yet been addressed, while iron sulfides are very well known as ubiquitous mineral precursors and energy sources for chemolithotrophic metabolism.

There are two major types of POMs, namely isopoly- and heteropolyoxoanions with the general formulas [M_m_O_y_]^n-^ and [X_x_M_m_O_y_]^n-^, respectively, where M represents the addenda atom, which is mostly restricted to tungsten, molybdenum and vanadium, and X the central heteroatom, which can be almost any other element. While there is a number of review papers and chapters reporting molybdenum and vanadium biochemistry and microbiological activities connected with these elements (Rehder, 1992, 2015; Winter and Moore, 2009; Hu and Ribbe, 2016; Leimkühler and Iobbi-Nivol, 2016), tungsten appears especially interesting, as there is no microbial respiration utilizing tungsten as terminal electron acceptor or primarily electron donor described in nature. Tungsten is the heaviest metal with various biological functions, especially in thermophilic archaea (Chan et al., 1995; Kletzin and Adams, 1996; Holden and Adams, 2003; Andreesen and Makdessi, 2008), but functional tungsten-based microbial respiration has not yet been observed.

Here, we present our observations on elemental ultrastructural analysis and nanoscale tungsten-microbial interface of the extreme thermoacidophile *M. sedula* cultivated with tungsten POM (K_6_[P_2_W_18_O_62_] or W-POM). Investigating whether complex inorganic systems based upon tungsten POM clusters can sustain chemolithotrophic growth of *M. sedula*, generate cellular proliferation and division, we provide our observations on the fate of the tungsten (W) during interactions of W-POM with *M. sedula*. We show that the use of tungsten-based inorganic POM clusters allows the possibility of incorporating tungsten redox heterogenous species by microbial cells.

## Materials and Methods

### Strain and Media Composition

*Metallosphaera sedula* (DSMZ 5348) cultures were grown aerobically in DSMZ88 *Sulfolobus* medium containing 1.3 g (NH4)_2_SO_4_, 0.28 g KH_2_PO_4_, 0.25 g MgSO_4_⋅7H_2_O, 0.07 g CaCl_2_⋅2H_2_O, and 0.02 g FeCl_3_⋅6H_2_O dissolved in 1 L of water. After autoclaving, Allen’s trace elements solution was added to 1 L media resulting in 1.80 mg MnCl_2_⋅4H_2_O, 4.50 mg Na_2_B_4_O_7_⋅10H_2_O, 0.22 mg ZnSO_4_⋅7H_2_O, 0.05 mg CuCl_2_⋅2H_2_O, 0.03 mg Na_2_MoO_4_⋅2H_2_O, 0.03 mg VSO_4_⋅2H_2_O, and 0.01 mg CoSO_4_ final concentration. The pH was adjusted to 2.0 with 10 N H_2_SO_4_.

### Cultivation Setup

Cultivation of *M. sedula* was performed as described before (Kölbl et al., 2017) in 1L glassblower modified Schott-bottle bioreactors (Duran DWK Life Sciences GmbH, Wertheim/Main, Germany), equipped with a thermocouple connected to a heating and magnetic stirring plate (IKA RCT Standard/IKA C-MAG HS10, Lab Logistics Group GmbH, Meckenheim, Germany) for temperature and agitation control. The bioreactors were equipped with three 10 mL graduated glass pipettes, permitting carbon dioxide and air gassing [with the gas flow of 9 mL min^-1^, adjusted to five bubbles s^-1^ by using 8 mm valves (Serto, Frauenfeld, Switzerland)] and sampling of culture, respectively. The graduated pipettes used for gassing were connected by silicon tubing to sterile 0.2 μm filters (Millex-FG Vent filter unit, Millipore, Billerica, United States). The graduated pipettes used for sampling were equipped with a Luer-lock system in order to permit sampling with sterile syringes (Soft-Ject, Henke Sass Wolf, Tuttlingen, Germany). The offgas was forced to exit via a water-cooled condenser (Ochs GmbH, Bovenden, Germany). For the cultivations of *M. sedula* at 73°C the temperature inside the bioreactors was controlled by electronic thermocouple via the heating and magnetic stirring plates. Cultures of *M. sedula* were supplemented with 10 g/liter of the Dawson-type tungsten-bearing POM W-POM (K_6_[α-P_2_W_18_O_62_]⋅*n*H_2_O) (Contant et al., 2007), preliminary temperature sterilized during autoclavation at 121°C for 20 min. Abiotic controls consisting of un-inoculated culture media supplemented with sterilized W-POM were included in all the experiments. Growth of cells was monitored by phase contrast/epifluorescence microscopy. To visualize wiggling cells, a modified “DAPI” (4′-6′- Diamidino-2-phenylindole) staining was used (Huber et al., 1985); afterward the cells were observed and recorded with ProgRes^®^ MF cool camera (Jenoptik) mounted on Nikon eclipse 50i microscope, equipped with F36-500 Bandpass Filters (ex, 377/50 nm; em, 447/60 nm).

### ESI-MS Characterization of W-POM Stability

Electrospray ionization mass spectrometry (ESI-MS) was used to characterize the stability of W-POM and the presence of the intact high mass tungsten oxo-clusters in this compound. The stability of W-POM compound was subject to investigation with ESI-MS after 21 days of cultivation with *M. sedula* in aqueous buffer solution. Biogenic samples of growth media supplemented with W-POM and inoculated with *M. sedula* and corresponding abiotic samples comprised of un-inoculated growth media supplemented with W-POM were collected at “0” time point and after 21 days of cultivation, filter-sterilized with 0.2 μm filters (Millipore, United States) and ESI-MS spectra were recorded.

### Scanning Electron Microscopy With Energy Dispersive Spectroscopy (SEM/EDS)

Cells of *M. sedula* grown on W-POM were harvested after 21 days of cultivation and were freshly prepared for electron microscopy by fixing in a solution of 1 vol.% glutaraldehyde in Na-Cacodylate buffer. Samples were dehydrated in a graded series of ethanol solutions and dried chemically using Hexamethyldisilazan (HMDS). Fixed samples were mounted on aluminum stubs, sputter-coated with a thin Au/Pd layer (Laurell WS-650-23 spincoater) and examined with a Zeiss Supra 55 VP scanning electron microscope (SEM), operated at 5 kV and equipped with a spectroscope of dispersive energy (EDS), which was used for imaging and elemental analysis of precipitates. Abiotic controls consisting of un-inoculated culture media were included in all the experiments. The acceleration voltage applied was 5 kV and the EDS analyses were performed with a 120 μm aperture and a counting time of 50 s. In order to control the beam parameters, cobalt was used as a standard. Conventional ZAF matrix correction was used to calculate the final composition from the measured X-ray intensities. All the sample spots investigated by EDS were chosen randomly and each spot was measured three times.

### Focused Ion Beam (FIB) Sample Preparation

Sample preparation for transmission electron microscopy (TEM) has been performed by focused ion beam (FIB) sputtering using a FEI Quanta 3D FEG instrument, equipped with an electron column hosting a field-emission electron source (Schottky emitter) and an ion column hosting a Ga-liquid metal ion source (LMIS). Sputtering progress has been monitored by electron beam (EB) induced secondary electron (SE) imaging at EB settings of 5 keV accelerating voltage, using standard mode spot number 3.5 and a 30 micrometer SEM aperture as probe current settings (yielding approximately 15 pA probe current). Before sputtering, a Pt layer (length × width × height = 8 × 3 × 3 micrometer) was deposited onto the cells of *M. sedula* by applying FIB Pt deposition at 16 kV IB acceleration voltage and 50 pA IB current for 5 min at the start, and continued after increasing IB current to 150 pA. The deposited nanocrystalline Pt served as protection layer during subsequent preparation steps. Using 30 kV FIB accelerating voltage and 7 nA IB current regular cross sections have been sputtered at both sides of the Pt top layer while the beam incidence was perpendicular to the substrate surface. All sections were performed with IB scanning directed toward the TEM foil, which represented the end point of each sputtering step. Before foil extraction from the sample, two cleaning cross sections have been sputtered at each side of the foil using 30 kV IB accelerating voltage, and IB currents of 5 and 1 nA, respectively. The three-micrometer thick foil has been transferred to an Omniprobe Cu TEM grid using an Omniprobe 100.7 micromanipulator for *in situ* lift-out. The sample has been temporarily mounted to a W-needle, and finally attached to the Cu TEM grid by Pt deposition at IB 30 kV and an IB probe current 100 pA. After the foil transfer to the TEM grid, several final thinning steps by cleaning cross sections have been performed alternately at both foil surfaces using successively lower IB probe current (300, 100, 50, and 10 pA) at successively smaller IB incidence angles (±1.5, 1.2, 1 degrees, respectively). The beam incidence direction therefore was close to parallel to the foil plane. Between each sputtering step the stage has been rotated by 180° in order to monitor the foil surface during sputtering and in order to achieve a perfectly central cut through the cells by reaching the maximum cell diameters. SE image contrast between the Pt top layer (higher signal intensity) and the cells (lower signal intensity) allowed monitoring the sputtering sites and the cells behavior during sputtering. Only a window area of the foil was completely finally thinned, whereas marginal parts of the foil had been left thicker to form a stabilizing frame. The obtained thinned foil area has finally been thinned to 55–65 nm thickness measured at the Pt top layer, but was slightly thinner in areas below the Pt top layer. The FIB preparation procedure led to the reduction of the maximum cell diameters, while Pt-deposition caused some flattening of the initially spheroidal cells. On the other hand, a slight off-center position of the cell cross-section could also lead to a reduction in the cross-section area of the cells.

### High Resolution Scanning Transmission Electron Microscopy (STEM) Imaging and EDX Investigations

Conventional and analytical TEM was performed using a FEI Tecnai F20 microscope operated at 200 kV under cryo conditions. The microscope is suited with a Schottky emitter, a monochromator, a post-column high resolution Gatan Imaging Filter (GIF) and a Si(Li) X-ray detector with ultrathin window. Energy filtered transmission electron microscopy (EFTEM), scanning transmission electron microscopy (STEM), and analytical spectroscopy by using electron energy loss (EELS) and dispersive X-ray (EDX) were carried out for different areas of *M. sedula* cells. For each area jump ratio images of the following elements: W, P, C, O, S, and Fe as well as EELS and EDX spectra from representative nano-particles on the cell surface and inside the cells have been acquired. EDX spectra were recorded in STEM mode, with a specimen diameter of about 1nm. The images and spectra were processed with Gatan’s Digital Micrograph being corrected for dark current and gain variations. Element quantification for both EELS and EDX spectra was performed by using the k-factor method (Hofer, 1991; Hofer and Kothleitner, 1993; Albu et al., 2016).

### Energy Loss Near Edge Spectra (ELNES) Simulations

The experimental ELNES spectra recorded from tungsten M-4,5 edges were compared to spectra simulated with the automated real space multiple scattering (RSMS) code FEFF 9.9.1 (Ankudinov and Ravel, 1998; Rehr et al., 2010) that aims to calculate measured spectra within the experimental accuracy (Moreno et al., 2007). Self-consistent muffin-tin potentials were used, obtained from Hedin-Lundqvist self-energies (Hedin and Lundqvist, 1971). Core-hole effects resulting from core-level excitation were treated using the final-state rule. Self-consistent field calculations were done on clusters of approximately 30 atoms. For a better comparison with experimental spectra the simulations have been broadened by a Lorentzian distribution according to the energy resolution displayed by the FWHM of the zero loss peak.

## Results

### Cultivation and Microbial–Metal Interactions

**FIGURE 1:**
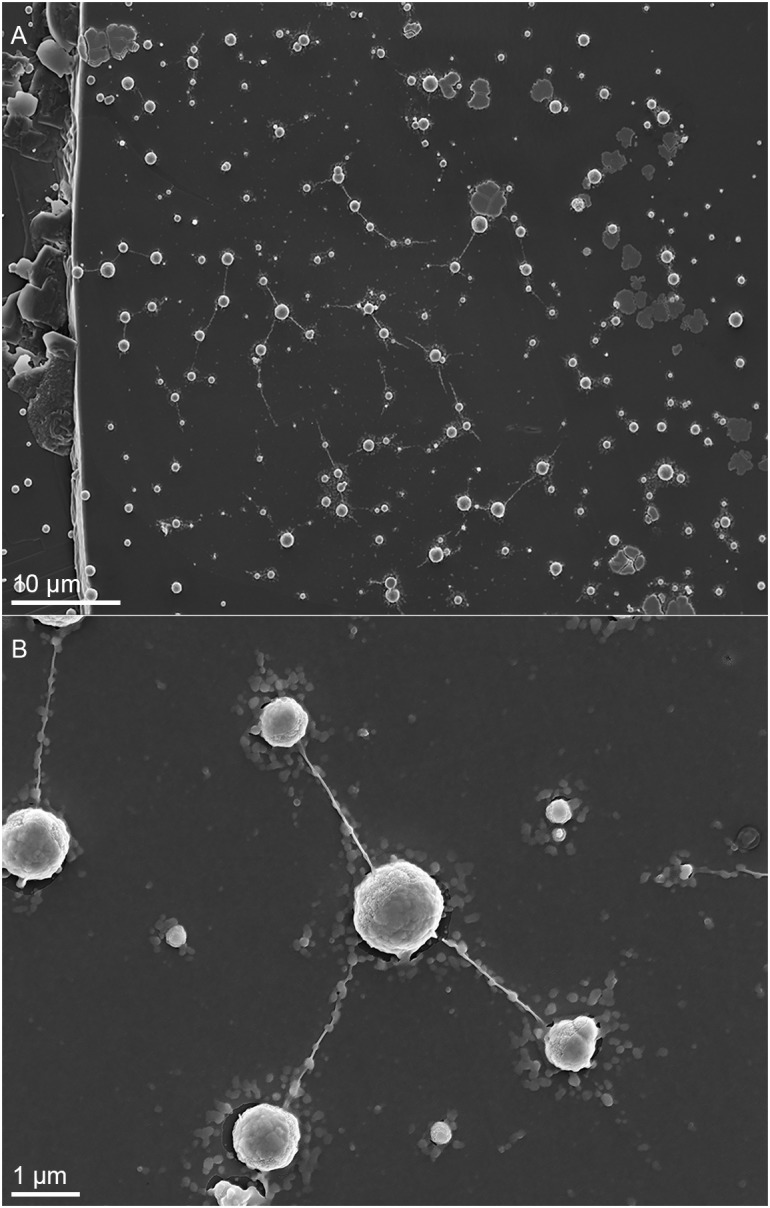
Scanning electron microscopy (SEM) images showing cells of *M. sedula* after 21 days of cultivation with W-POM. **(A)** SEM image of colonies of *M. sedula* cells showing single cells of *M. sedula* connected by means of extracellular extensions. **(B)** Magnified SEM of the cellular assemblage, revealing the network of filamentous membrane extensions (the diameter in a size range of 10–30 nm) between the neighborhood cells of *M. sedula*. A number of nano-sized vesicles are localized in the areas surrounding the single cells and along the extracellular extensions.

**FIGURE 2:**
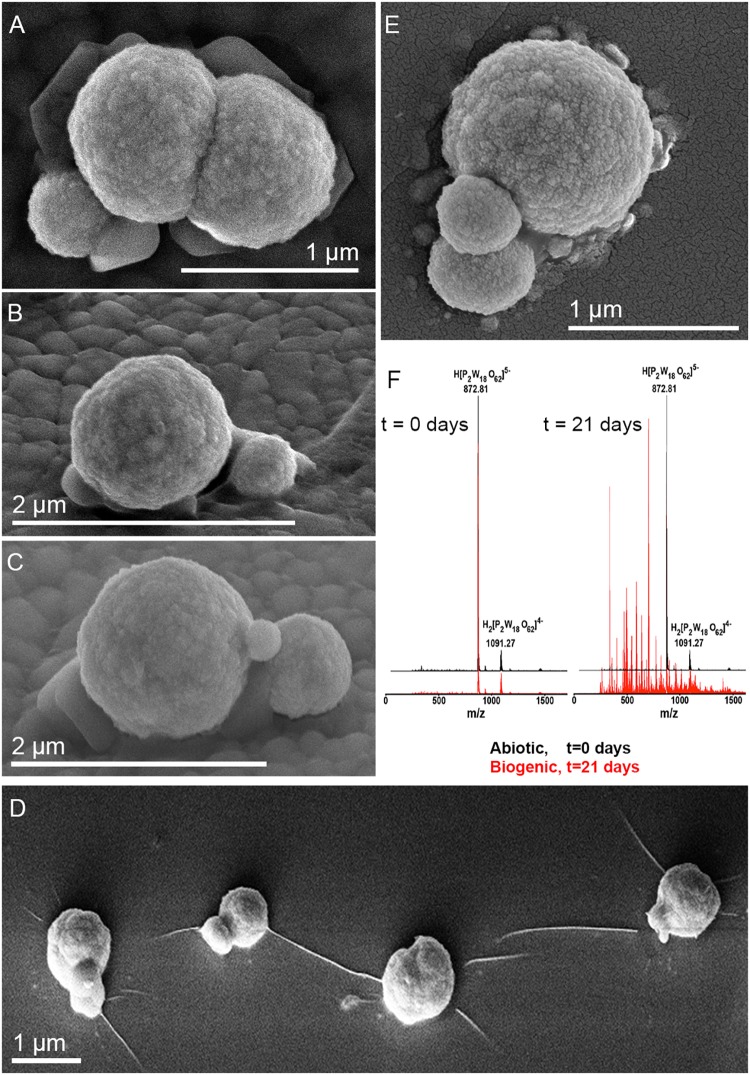
Scanning electron microscope images of *M. sedula* cells and ESI-MS patters after 21 days of cultivation with W-POM. **(A–E)** SEM images showing various assemblages of dividing *M. sedula* cells and cells forming budding vesicles. **(F)** Experimental ESI-MS analysis of biogenic cultures of *M. sedula* (red pattern) and corresponding abiotic control comprised of non-inoculated growth medium (black patter) at the “0” time point and after 21 days of cultivation with W-POM.

*Metallosphaera sedula* was cultivated in DSMZ88 *Sulfolobus* medium supplemented with W-POM (Supplementary Figure 1) and microbial–metal interactions were examined. Cultures of *M. sedula* reached maximum cell density of 1.43 × 10^7^ cells/ml after 21 days of CO_2_-supplemented cultivation with W-POM as the sole energy source (Table 1). In comparison with mineral-supplemented autotrophic growth, *M. sedula* showed substantially reduced cell densities when cultivated on W-POM in basal salts medium (Table 1), whereas the drop in cell densities was observed when no W-POM and any other energy source was added to the cultivation medium (Table 1). Studies with scanning electron microscopy (SEM) showed colonies of *M. sedula* cells after 21 days of CO_2_-supplemented cultivation with W-POM (Figure 1A,B); frequently, within these colonies single cells were connected with each other by means of extracellular appendages (Figure 1A,B and Supplementary Figure 2). These appeared as filamentous extensions networking the neighborhood cells of *M. sedula*. A number of nano-sized vesicles were clearly represented in the areas surrounding the single cells and along the extracellular appendages (Figure 1B and Supplementary Figure 2). In addition, we were able to capture the cells of *M. sedula* with different morphologies, which correspond to different stages of “cell cycle”: dividing cells and cells forming budding vesicles were clearly recognized too (Figure 2A–E).

**Table 1:** Cell densities of *M. sedula* at “0” time point and after 21 days of cultivation with W-POM.

Cultivation time	0 day	21 days
	
	Cell density [cells/ml]	
W-POM	4,36E+06 ± 3,97E+05	1,43E+07 ± 1,09E+05
P-MRS	5,34E+06 ± 2,68E+06	4,95E+08 ± 4,35E+08
Pyrite	6,57E+06 ± 3,08E+05	1,55E+09 ± 2,5E+08
No. W-POM added	6,95E+06 ± 7,85E+05	9,53E+04 ± 2,90E+04


*Mean and standard deviation of *n* = 3 biological replicates are represented. When no W-POM was added, cultivation medium was inoculated with cells of *M. sedula* without additional energy source. For comparison, cell densities of *M. sedula* grown on pyrite FeS_*2*_ and multimetallic Martian Regolith Simulant P-MRS (Kölbl et al., 2017) are provided.*

**FIGURE 3:**
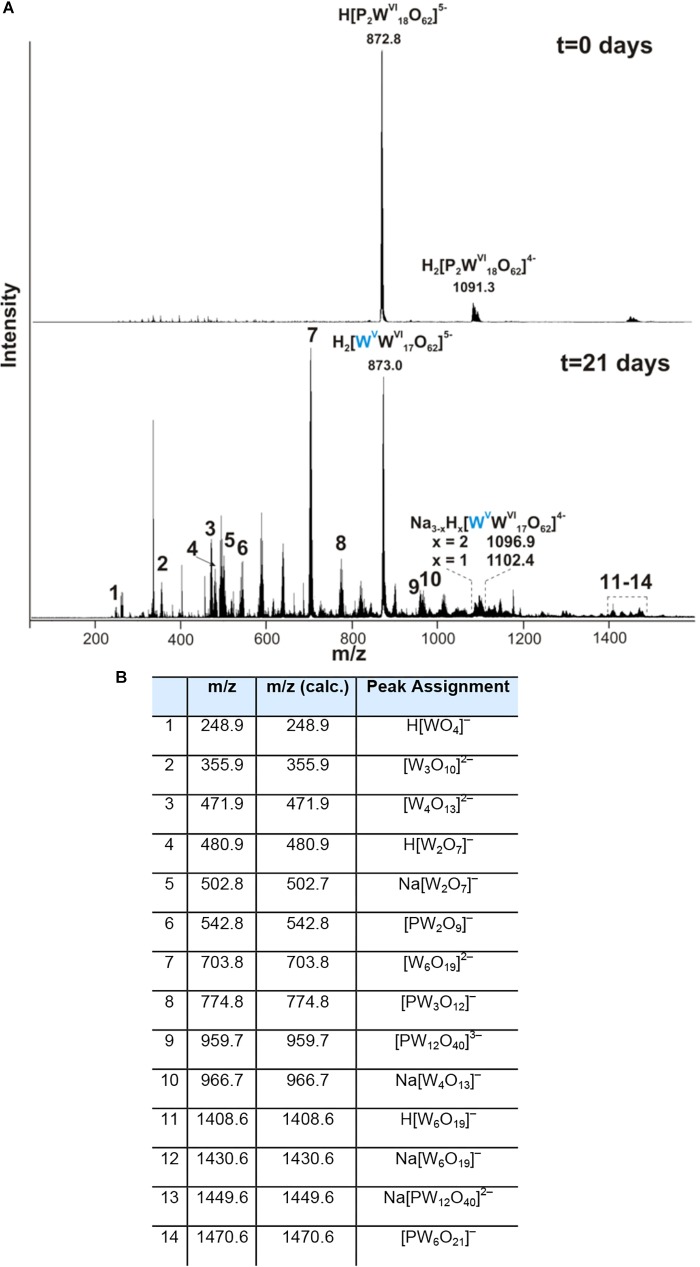
Negative ion-mode ESI-MS spectrum after 0 and 21 days of cultivation with W-POM in mixed H_2_O/CH_3_CN **(A)** and corresponding species assigned to the peaks in the ESI-MS spectrum in panel **(A,B)**.

**FIGURE 4:**
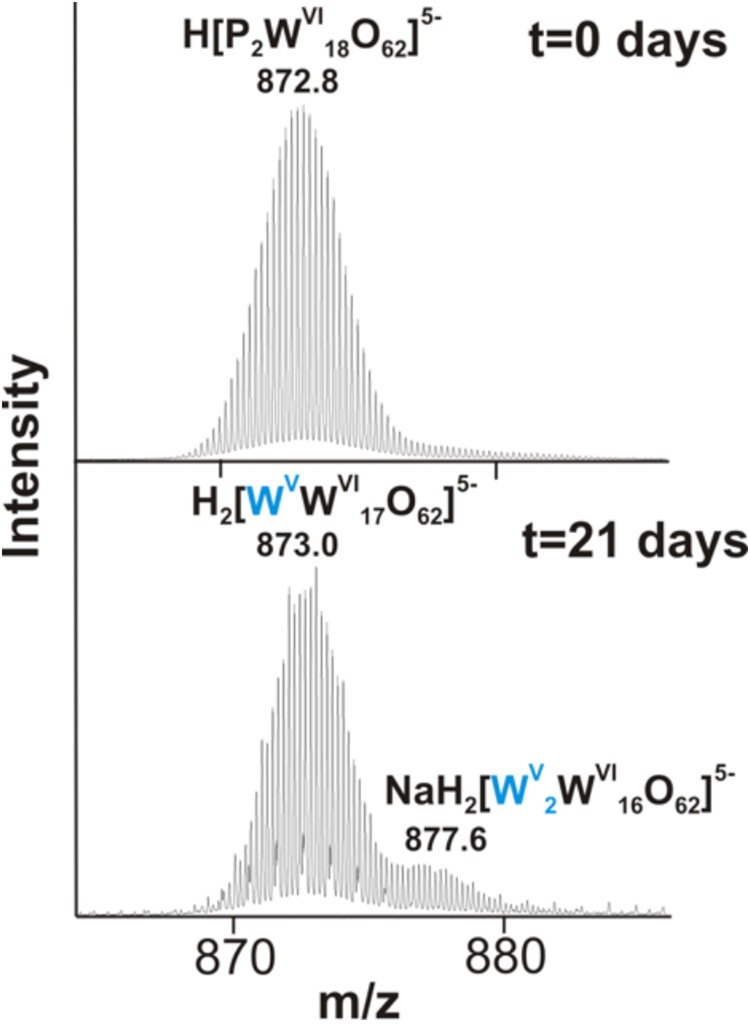
The region of 860–890 m/z from the negative ion-mode ESI-MS spectra after 0 and 21 days of *M. sedula* cultivation with W-POM (Figure 3).

We further examined the interaction of the cells of *M. sedula* with the W-POM macromolecular clusters, applying ESI-MS-assisted characterization of the W-POM compound. Figure 2F shows the presence of the intact clusters in the W-POM compound [high mass tungsten oxo-clusters H_2_[P_2_W_18_O_62_]^4-^ (1091.27) and H[P_2_W_18_O_62_]^5-^ (872.81)] at zero time point of cultivation in both biogenic and abiotic samples. The spectra of biogenic cultures of *M. sedula* displayed a quite complex fragmentation pattern with the appearance of lower mass fragment ions after 21 days of cultivation with W-POM, while the spectra of abiotic samples after 21 days confirmed the presence of the intact clusters in the W-POM compound and the absence of fragment ions with lower mass (Figure 2F). Thus, the appearance of low mass tungsten oxo-fragments was detected in the harvested cultures of *M. sedula* by ESI-MS, and these decomposed low mass tungsten oxo-fragments were not presented in corresponding abiotic control (Figure 2F). Our extremely sensitive ESI-MS analysis further permitted the detailed investigations of the obtained spectrum after 21 days of *M. sedula* cultivation with W-POM. This spectra reveals complex character (Figure 3A) and is dominated by peaks which can be assigned to different decomposition fragments of W-POM ([W_m_O_3m+1_]^2-^, H[W_m_O_3m+1_]^-^ or Na[W_m_O_3m+1_]^-^, where m = 1 – 4; [PW_12-n_O_39-3n_]^-^, where *n* = 2, 3, 6 see Figure 3B). Apart from this, the main difference from spectrum recorded at zero time point of cultivation is the absence of fully oxidized Dawson polyoxotungstate (872.8 m/z) and presence of 1-(873.0 m/z) and 2-electron (877.6 m/z) reduced anions (as visible for the most intensive peaks in Figure 4). The signal from the spectra recorded after 21 days of cultivation exposes different envelope distribution as well as an additional peak area for 2 –electron reduced anions (877.6 m/z) (Figure 4).

To improve our understanding of *M. sedula*-W-POM interactions, the cell surface of *M. sedula* was examined with SEM coupled with Energy Dispersive Spectroscopy (SEM-EDS) analysis. SEM-EDS analysis indicated the cell surface biomineralization with tungsten (Supplementary Figure 2 and Supplementary Table S1). Inorganic ions relieved in course of W-POM biotransformation tended to accumulate on the surface of *M. sedula* cells, forming mineral precipitates on its surface-layer (S-layer). Various mineral precipitates were reported earlier on the S-layer of other biomineralizing extremophilic archaea and bacteria (Orange et al., 2011, 2014; Oggerin et al., 2013; Sánchez-Román et al., 2015; Kish et al., 2016). The EDS spectrum of mineralized cells of *M. sedula* displayed strong dominated C peak along with less intensive peaks of O and W (Supplementary Figure 2 and Supplementary Table S1). Along with the colonies of *M. sedula* (Figure 1 and Supplementary Figures 3, 4), we observed a deposition of needle-like and plate-like structures enriched with W signal as detected by SEM-EDS analysis in samples of *M. sedula* cultures (Supplementary Figure 5 and Supplementary Table S2). These needle- and plate-like deposits, which are absent in abiotic control (Supplementary Figure 6D), may represent W-POM decomposition products and/or products of *M. sedula* metabolic activity. Areas with carbon-enriched deposits occurred as well, which can be inferred to be exopolymeric substances and/or cell debris protruding the surface of crystalline material (Supplementary Figure 6 and Supplementary Table S3).

### Ultrastructural Analysis of Biomineralized *M. sedula*

**FIGURE 5:**
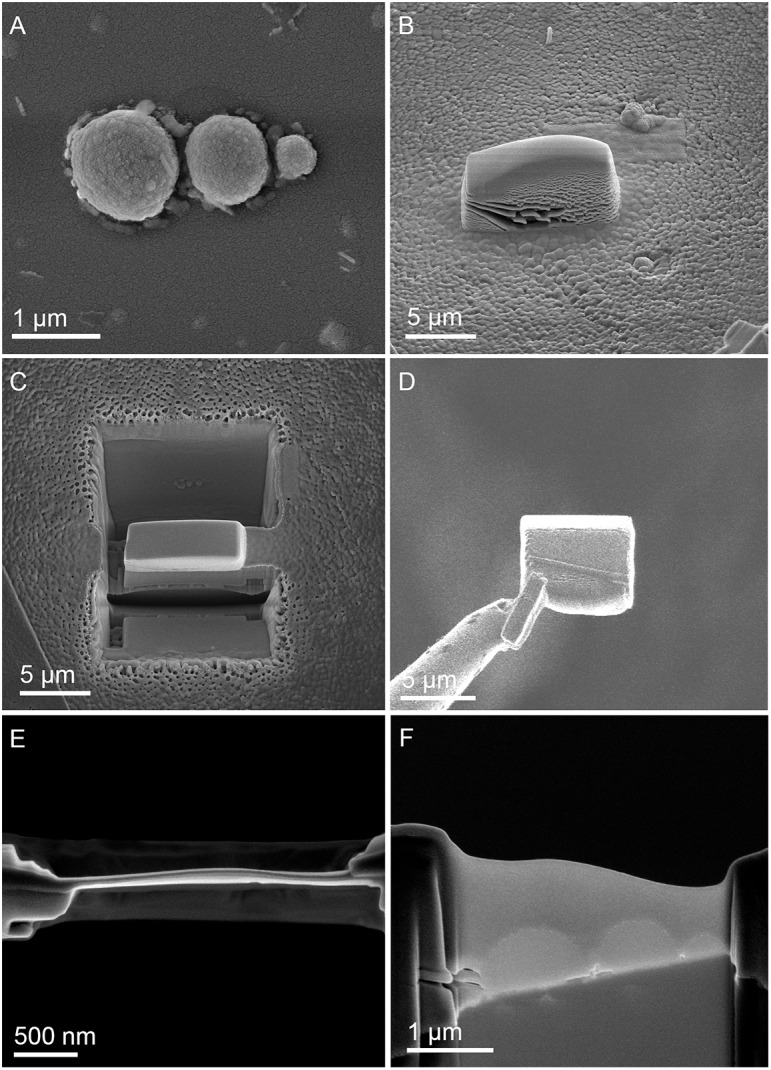
Focused Ion Beam (FIB) assisted preparation of thin lamellae of *M. sedula* cultivated on W-POM documented by electron beam induced SEM images. **(A)** SEM image of a cell assemblage of *M. sedula* used for milling. **(B)** 3 μm thick Pt deposition layer covering cells of *M. sedula*. **(C)** FIB removal of material at both sides of the Pt layer viewed perpendicular to the substrate surface. **(D)** Transfer of the 2.5 μm thick lamella from the micromanipulator needle (left) to the Cu TEM grid. **(E)** Finally thinned lamella showing the Pt layer in top-view. **(F)** Side-view of the finally thinned lamella showing the three flattened cells covered by a Pt layer.

**FIGURE 6:**
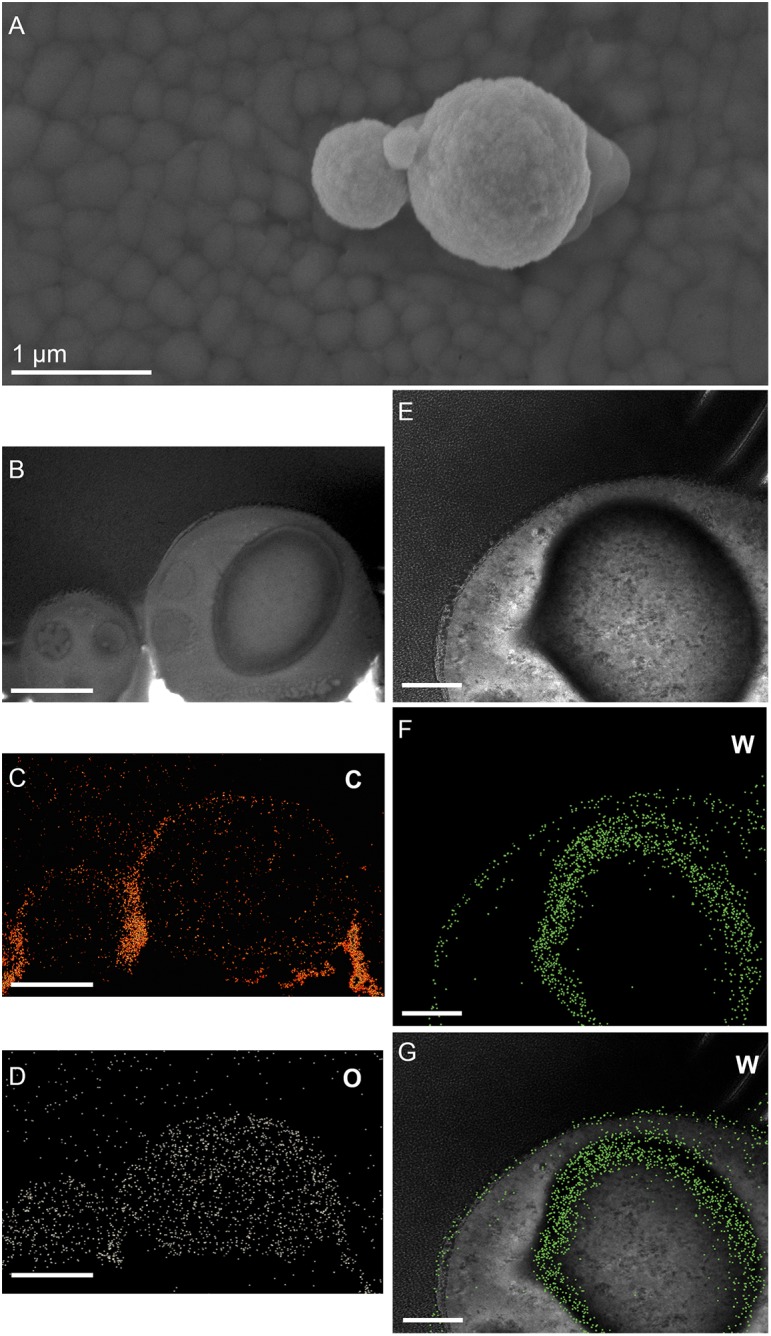
Elemental ultrastructural analysis of cells of *M. sedula* after 21 days of cultivation with W-POM. **(A)** Transmission electron microscopy image showing an assemblage of the cells of *M. sedula* with the selected area used for energy-filtered transmission electron microscopy (EFTEM) analysis. **(B–E)** Corresponding tungsten (W), oxygen (O), carbon (C), and phosphorous (P) elemental maps. **(F)** The high angular annular dark field (HAADF) scanning TEM (STEM) image of a cell fragment of *M. sedula* with the depicted areas for W M_3,4,5_–edge core electron energy loss spectra (EELS) analysis. **(G)** Corresponding representative W M_3,4,5_–edge core EELS of W-POM and EELS acquired from the areas depicted in panel **(F)**.

**FIGURE 7:**
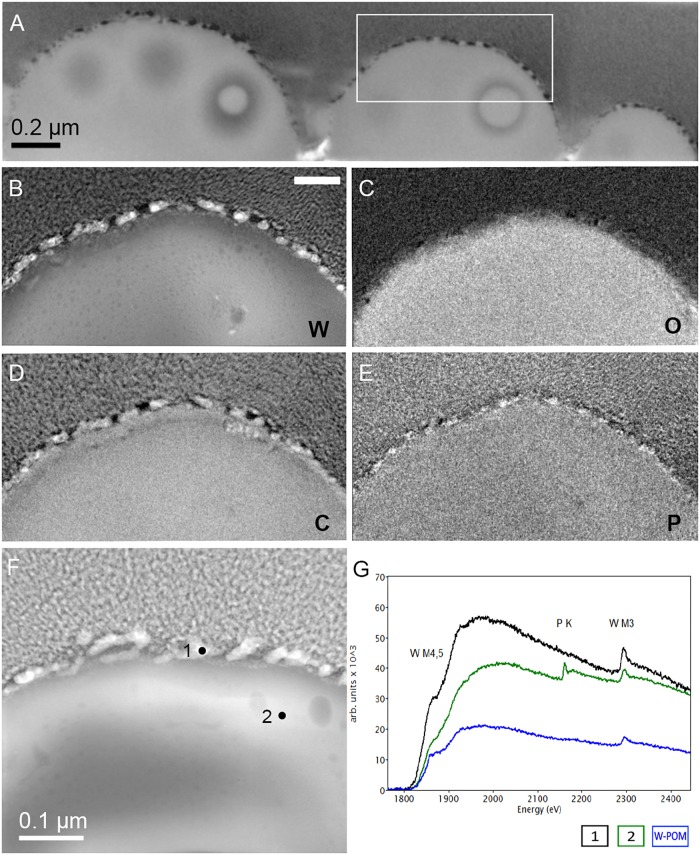
Additional elemental ultrastructural analysis of cells of *M. sedula* after 21 days of cultivation with W-POM. **(A)** Scanning electron microscopy (SEM) image showing an assemblage of the cells of *M. sedula* used for transmission electron microscopy (TEM) analysis. **(B)** TEM image showing an assemblage of the cells of *M. sedula* **(C,D)** Corresponding carbon (C) and oxygen (O) elemental maps. **(E)** Magnified TEM image of a cell of *M. sedula*. **(F,G)** Corresponding tungsten (W) elemental map and overlaid image. Scale bars are 1 μm.

To further characterize microbial-mineral interface, we performed ultrastructural analysis of cells of *M. sedula* cultivated with W-POM. Tungsten-biomineralized cells of *M. sedula* represented a kind of hard and brittle biological material that is difficult to cut with a diamond knife in conventional ultramicrotome procedures. Therefore, in order to overcome this limiting factor, a FIB-SEM was applied to prepare foil samples for the subsequent ultrastructural analysis at high resolution (Figure 5). Further elemental ultrastructural analysis enabled us to verify the content and localization of metals at the surface of S-layer and within the cells of *M. sedula* (Figure 6, 7 and Supplementary Figure 7). The following observations were made by using energy filtered TEM (EFTEM) and high angular annular dark field (HAADF) imaging and energy-dispersive X-ray spectroscopy (EDX) as well as electron energy loss spectroscopy (EELS) analyses performed by STEM:

(1) the elemental distribution maps showed abundant tungsten content on the cell surface (S-layer) of *M. sedula*;
(2) tungsten was deposited as nano-globules in a size range of 10–50 nm on the cell surface massively encrusting *M. sedula* cells;
(3) intracellular accumulates of tungsten of cluster size (couple of atoms) were associated with the round-shaped granules (storage bodies and depots) of nanometer-range size and were distributed through the cell;
(4) oxygen and carbon were evenly represented giving strong intracellular signals which likely arose from organic content (e.g., proteins) present in *M. sedula* cells;
(5) phosphorus signal was more pronounced in the area of membrane localization (S-layer);
(6) no iron and sulfur signal was detected across the whole cell of *M. sedula*, which is in conjunction with the applied cultivation medium where W-POM was used as the sole energy source (Supplementary Figure 8).

### Tungsten Deposits in *M. sedula*

The electron energy loss (EEL) spectra of W-M4,5, and W-M3 edges were acquired from W-POM, crystalline S-layer deposits and intracellular nanoparticles (Figure 6G). The characteristic sharpness of W-M4,5 edge in W-POM is not anymore represented in EELS and ELNES W-M4,5 edge acquired from the biogenic tungsten-bearing structures of the cell surface and intracellular content of *M. sedula*, suggesting that the biotransformation of W-POM occurred and biogenic incorporated tungsten-bearing material is chemically different from initial W-POM compound (Figure 6G, 7E).

**FIGURE 8:**
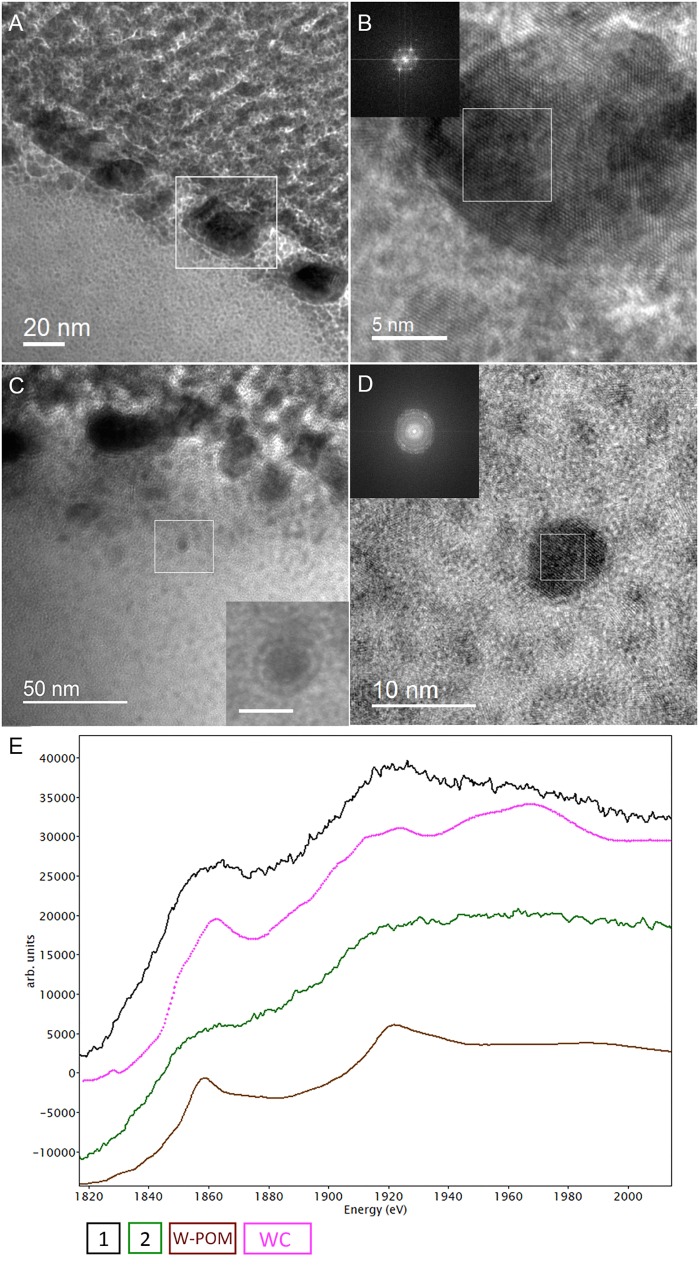
Analytical spectroscopy of biogenic tungsten deposits in the cells of *M. sedula* after 21 days of cultivation with W-POM. **(A)** High-resolution TEM (HR-TEM) image of S-layer fragment of *M. sedula* cell. **(B)** HR-TEM and Fast Fourier Transform (FFT) acquired from the tungsten crystalline cell surface deposits labeled in panel **(A)**. **(C)** HR-TEM image of the intracellular part of *M. sedula* cell; inlet represents magnified labeled area showing that intracellular nanoparticle is surrounded by electron dense layer which may suggest the presence of membrane; **(D)** HR-TEM and FFT obtained from intracellular nanoparticle labeled in panel **(C)**. Insets in panel **(B,D)** represent diffraction patterns consistent with the tungsten carbide WC and W_2_C phases. **(E)** Comparison of Energy Los Near Edge Spectra (ELNES) recorded from W-M4,5 edge with the reference spectra from a WC phase (pink line) and simulations of W-containing phases of W-POM (brown line) and of the areas depicted in Figure 5F (black and green lines).

The cell ultrastructure of *M. sedula* was further examined by high-resolution TEM (HR-TEM) performed on thin foils (ca. 60 nm thickness) preliminary produced by FIB milling (Figure 5). HR-TEM analysis revealed a crystalline microstructure of tungsten-bearing deposits over S-layer of the cells of *M. sedula* with characteristic lattice fringes observed in crystalline materials (Figure 8 and Supplementary Figures 9, 10). This crystalline tungsten-bearing layer is comprised of globular deposits of up to 50 nm thickness which massively encrust the surface area of the cells of *M. sedula* (Figure 8A,B and Supplementary Figure 9). Apart from that, the intracellular accumulation of tungsten-bearing clusters was clearly observed during HR-TEM analysis (Figure 8C,D). High magnification HR-TEM image of one of such intracellular nanoglobules (Figure 8C, inlet) reveals adjacent electron-dense layer, which surrounds nanoglobule and is consistent with the presence of a membrane on this “tungstosome.” The electron diffraction patterns were collected from both surface crystalline layer and intracellular nanoinclusions (Figure 8 and Supplementary Figures 9, 10). All are consistent with the structure of tungsten carbides WC with hexagonal unit cell (*a* = 0.28946 nm, *b* = 0.28946 nm, *c* = 0.28576 nm) (Page et al., 2008) and W_2_C with trigonal unit cell (*a* = 0.5188 nm, *b* = 0.5188 nm, *c* = 0.47273 nm) (Kurlov and Gusev, 2007). HR-TEM analysis further revealed that W_2_C was predominant on the cell surface (Figure 8A,B and Supplementary Figure 10), while the intracellular nanoinclusions have a structure of WC – type (Figure 8C,D). Such intracellular accumulations of metal ions in storage granules constitute an additional metal–organic matter interface and may possibly serve as a storage pull of these elements inside the cell or/and as a detoxification depot. Interestingly, the EEL spectra number 2 point-acquired from intracellular region pointed on the HAADF image in Figure 6F indicates a sharp peak (Figure 6G) which can be inferred to be a phosphorus sequestration of heavy metals as an effective detoxifying mechanism frequently employed by metal tolerant microorganisms (Merroun, 2007; Gadd, 2010).

## Discussion

Many of important functional applications of POMs, e.g., as catalysts (Proust et al., 2008), biotechnological, biomedicinal, and nanotechnological agents (Yamase and Pope, 2002; Lv et al., 2012; Bijelic et al., 2018), and as electron repositories in battery cathodes (Wang et al., 2012), rely on their unique redox active properties. These molecules are often recognized as electron reservoirs due to their high capacity to bear and release electrons (Gumerova and Rompel, 2018). POMs are considered as a combination of weak Lewis bases due to their surface oxo ligands and Lewis acidic sites due to the addenda metal ions of the polyanion skeletons bearing unoccupied orbitals, such that the outside of POMs can also provide electrophilic sites (Wang and Yang, 2015). This dual nature of reduced POMs creates a possibility for electron hopping from one metal addendum ion to the next under the hydrothermal conditions. Such an electron circulation most probably involves the bridging oxo ligands participating in π-bonding with the reduced metal ion M^5+^ and its oxidized neighbor addendum M^6+^ (Wang et al., 2012; Gumerova and Rompel, 2018). Formation of reduced POMs species featuring the Wells–Dawson structure has been described in molecular solvents under the hydrothermal conditions as an overall one- or two-electron reduction step with intermediate multi-electron reduced anions [P_2_W_18_O_62_]*^n^*^-^ (*n* > 6), yielding the so-called green 1e- reduced species or blue 2e- reduced species.

Our ESI-MS analysis suggested the decomposition of W-POM macromolecular clusters on smaller oxo-fragments in the presence of *M. sedula* (Figure 2F, 3 and Supplementary Figure 11). The presence of these W-POM decomposition products was not observed in abiotic samples after 21 days of incubation in growth medium in the absence of *M. sedula* (Figure 2F). How would a microbe with metal-oxidizing metabolic activity decompose tungsten POM that contains tungsten in the highest oxidation state of 6^+^? Microorganisms such as metal oxidizers can utilize a mechanism to respire directly on electron flow under the conditions where electron donor is available (Choi and Sang, 2016; Stelmach et al., 2018). The cultivation and growth of *M. sedula* occurs in single medium of complex composition with a number of parameters (e.g., acidic pH, elevated temperature, ionic strength, pressures of gas phases), which can upraise the appearance of green or blue reduced W-POM species, affecting the ratio of addendum W^6+^/W^5+^ and thus stimulating electron circulation within one single molecule of W-POM. The source of the electrons for the reduction of Dawson polyoxotungstate at first sight is not obvious; here, we present several possible scenarios which may likely occur in our system. One of the possible plausible explanations is that the generation of reduced W-POM derivatives can be attributed to electrons received from either trace contaminants in the air supply or from the degradation of storage compounds in the cells of *M. sedula* used for inoculation. Considering the oxidizing power of P_2_W_18_O_62_^6-^ (Chen et al., 2018) and not fully oxidized elements in the cultivation media (Mn^2+^, Co^2+^, and V^4+^) along with hydrothermal condition, several additional reduction reactions can take place. It was already shown (Bai et al., 2008; Tian et al., 2015; Li and Huang, 2016) that POMs can oxidize even molecules with higher redox potentials under hydrothermal conditions, thus the trace amount of V^IV^O^2+^ (∼0.1 μM), Mn^2+^ (14 μM), and Co^2+^ (∼0.1 μM) in DSMZ88 *Sulfolobus* medium used for *M. sedula* cultivation (see section “Materials and Methods”) can induce the electron flow to W-POM, resulting in appearance of blue reduced W-POM [P_2_W^V^_n_W^V I^_(18-n)_O_62_^(6+n)-^] species:

**Table:** 

Oxidation	V^IV^O^2+^ + H_2_O – 1 e^–^ → V^V^O^2+^ + 2H^+^ Mn^2+^ + H_2_O – 2 e^–^ → MnO_2_ + 4H^+^ Co^2+^ – 1 e^–^ → Co^3+^
Reduction	P_2_W^V I^_18_O_62_^6–^ + n e^–^ → P_2_W^V^_n_W^V I^_(18-n)_O_62_^(6+n)–^


Moreover, the high content of ammonium cation (10 mM) in the DSMZ88 *Sulfolobus* medium used for *M. sedula* cultivation provides potential source for hydrothermal oxidation of nitrogen (Aymonier et al., 2000; Lam et al., 2008) catalyzed by Dawson polyoxotungstate, also resulting in appearance of blue reduced W-POM [P_2_W^V^_n_W^V I^_(18-n)_O_62_^(6+n)-^] species:

**Table:** 

Oxidation	2NH_4_^+^ – 6 e^–^ → N_2_(g) + 8H^+^
Reduction	P_2_W^V I^_18_O_62_^6–^ + n e^–^ → P_2_W^V^_n_W^V I^_(18-n)_O_62_^(6+n)–^


The specific coloration pattern of blue reduced W-POM [P_2_W^V^_n_W^V I^_(18-n)_O_62_^(6+n)-^] species in DSMZ88 *Sulfolobus* medium used for *M. sedula* cultivation is represented in Supplementary Figure 12A, while it is absent in intact synthesized powder of W-POM (Supplementary Figure 12B). Furthermore, our ESI-MS investigations also indicate the presence of blue 1- (873.0 m/z) and 2-electron (877.6 m/z) reduced anion species (Figure 3, 4 and Supplementary Figure 11). In this scenario, considering the multifactorial complexity and multicomponent character of the investigated system (pH, ionic strength, partial pressures of CO_2_ and air, temperature etc.), being highly soluble in water, K_6_[P_2_W_18_O_62_] can provide an environment where a variety of mass transport outcomes occur, e.g., electrons rapidly hopping within blue 1e- reduced and 2e- reduced species of W-POM may create a miniaturized electron flow.

The observed presence of extracellular filamentous appendages connecting the single cells of *M. sedula* grown on W-POM attracts a special attention (Figure 1 and Supplementary Figure 2). The cells and attached filamentous appendages were clearly resolved in this case. We have observed the formation of such cellular extensions explicitly in a situation with W-POM-based cultivation of *M. sedula.* None of the tested mineral substrates on which *M. sedula* usually respires (mineral ores and complex artificial mineral matrixes) gave a rise to the formation of similar cellular extensions. Microorganisms are recognized to produce cellular extensions for a diversity of functions, including long-range electron transfer via conductive extracellular filaments known as microbial nanowires or conductive pili (Wegener et al., 2015; Choi and Sang, 2016; Walker et al., 2018). The genome of *M. sedula* encodes the putative pili Pil/Fla operons, which are well conserved in the order Sulfolobales. The analysis of *M. sedula* genome revealed several gene clusters matching to the type II/IV/Fla systems (Auernik et al., 2008). Msed1324 to Msed1330 appear to encode seven flagellar proteins (FlaJ, FlaIH, FlaFG, and FlaB), while FlaIJ-like protein genes are encoded by Msed1197, Msed1198, Msed2104, Msed2105, Msed0650, Msed0651 and a prepilin/preflagellin peptidase gene (FlaK-like sequence) is located upstream of one of these FlaIJ pairs (Msed2090) (Auernik et al., 2008). For *M. sedula*, it remains still to be resolved whether the observed extracellular filaments are outer-membrane extensions and if they facilitate extracellular electron transport from inorganic electron-rich substrates or exchange of genetic material in between mother and daughter cells. The occurrence of these cellular extensions exceptionally in case with W-POM certainly supports the first possibility, e.g., that membrane filamentous extensions may represent a strategy of *M. sedula* for electron transport and energy circulation between the single cells. Although, electrophilic (“electron-loving”) properties of *M. sedula* were not shown yet, this is an interesting issue that deserves a special attention in the future. In this connection, the analysis of the protein content of the extracellular milieu, i.e., exoproteome might represent an efficient strategy to screen for putative redox exo-proteins which are expelled by the cells either in vesicles or/and are the parts of extracellular appendages. Knowledge of the mechanisms and structures of extracellular electron transfer which *M. sedula* may potentially utilize, e.g., the presence of soluble electron shuttles in EPS and/or conductive pili, can further facilitate the production of effective alternative electrofuels.

Our HR-TEM analysis and SAED patterns (Figure 8 and Supplementary Figure 10) show that the extreme thermoacidophile *M. sedula* mineralizes its S-layer via tungsten carbide-like compounds. The presence of tungsten carbide-like materials is rather unusual because WC is generally considered to form under much higher temperatures. Whether the observed biomineralization occurs via a direct precipitation of tungsten carbide from the cultivation medium or is coupled to active metabolic respiration is rather unresolved issue. Putatively it is possible that the accumulation of reduced W species might be utilized by *M. sedula* as a respiration source, although this kind of tungsten-based chemolithotrophy has not yet been demonstrated for any microbial entity. In this regard, the molecular machinery of *M. sedula* responsible for tungsten binding, acquisition and assimilation is a topic that requires more thorough analysis. This can be further effectively addressed by means of advanced synchrotron-assisted spectroscopic studies of the nanoscale interface between tungsten and *M. sedula*. Our results do not unravel the exact mechanism of W-POM biotransformation and tungsten carbide formation, but do indicate that *M. sedula* forms tungsten-bearing mineralized S-layer via encrusting with tungsten carbide-like compounds. The possibility for the formation of membrane-bound WC-like materials also might raise due to the fact that carboxyl COOH groups of the S-layer proteins frequently act as reactive sites and functional groups on the surface of microorganisms for the adsorbing metal cautions on the cell surface, thus generating WC-like bonding in case of freely available W ions in the solution. To determine the nature of W environment in these biogenic deposits further work might be concentrated on performing W μ-XANES in our samples.

## Conclusion

Our study motivates further experimental and theoretical work to build up a detailed understanding of the full functional molecular set up of life based on tungsten-bearing inorganic sources. This has significant implications for the exploration of new inorganic-based energy sources, plausible redox couples and their geobiochemical proxies as potential candidates for biosignatures. Here, of a special interest is a newly described tungsten-microbial biomineralized interface of *M. sedula*. While the use of morphological assessment and carbon-content evaluation may not fully be useful as a criterion for distinguishing biogenic from abiotic chemistries, metallo-organic interfaces can provide a helpful evidence to support a biological origin of examined samples. Our findings add tungsten-encrusted *M. sedula* to the growing records of biomineralized microbial species, among which archaea are rarely represented.

## Author Contributions

MA, AB, NG, LK, NC, and TM performed the experiments. TM and AB planned and designed the study. All authors made substantial contributions to the acquisition, analysis, and interpretation of the data described in this manuscript, reviewed the report, and approved the final version.

## Conflict of Interest Statement

The authors declare that the research was conducted in the absence of any commercial or financial relationships that could be construed as a potential conflict of interest.
