# Supplementary figures and images for: Nanoscale Tungsten-Microbial Interface of the Metal Immobilizing Thermoacidophilic Archaeon Metallosphaera sedula Cultivated With Tungsten Polyoxometalate
Source: Front Microbiol. 2019 Jun 7;10:1267. doi: 10.3389/fmicb.2019.01267 (PMC6593293; doi:10.3389/fmicb.2019.01267)

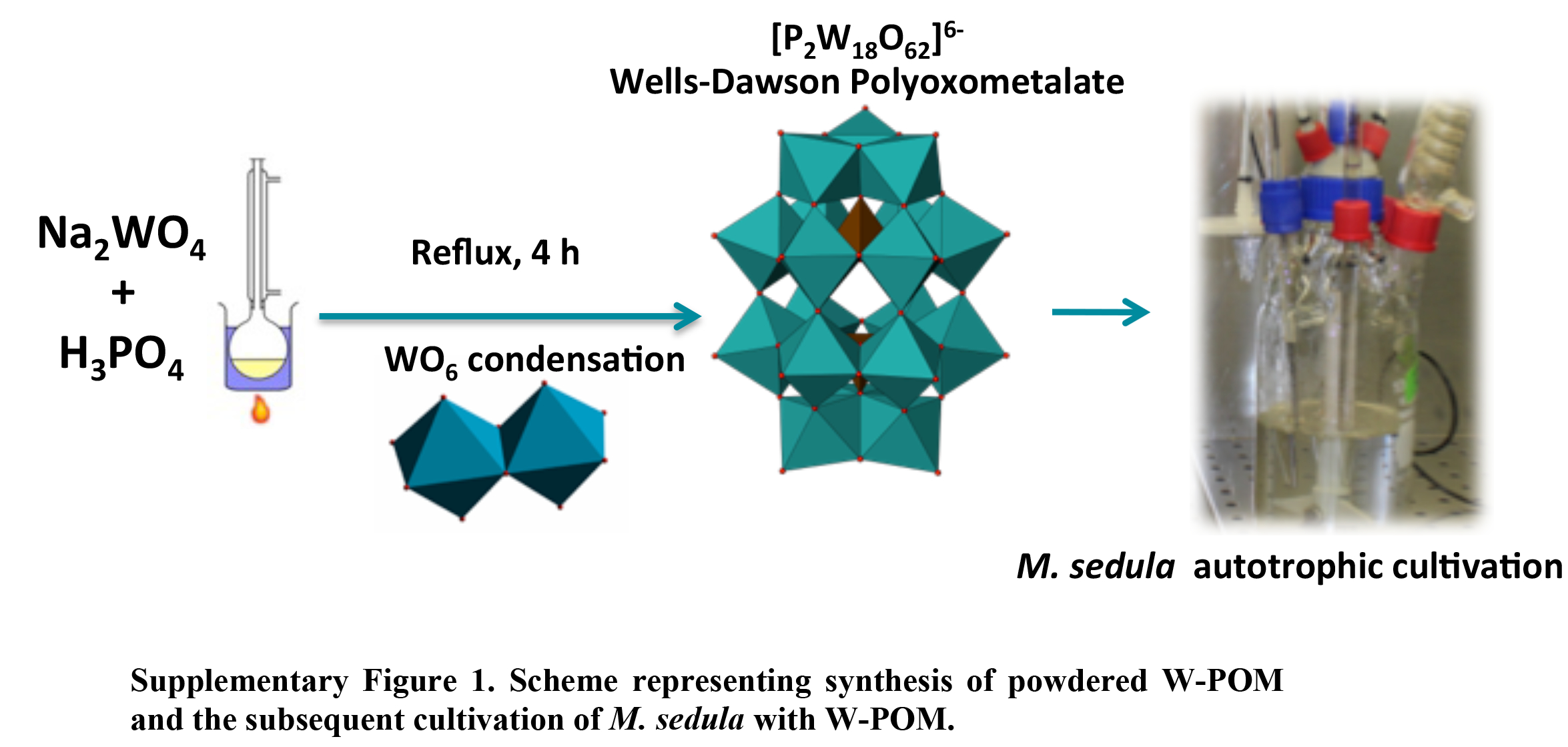

Supplement: Supplementary file 1 [file Image_1.JPEG]

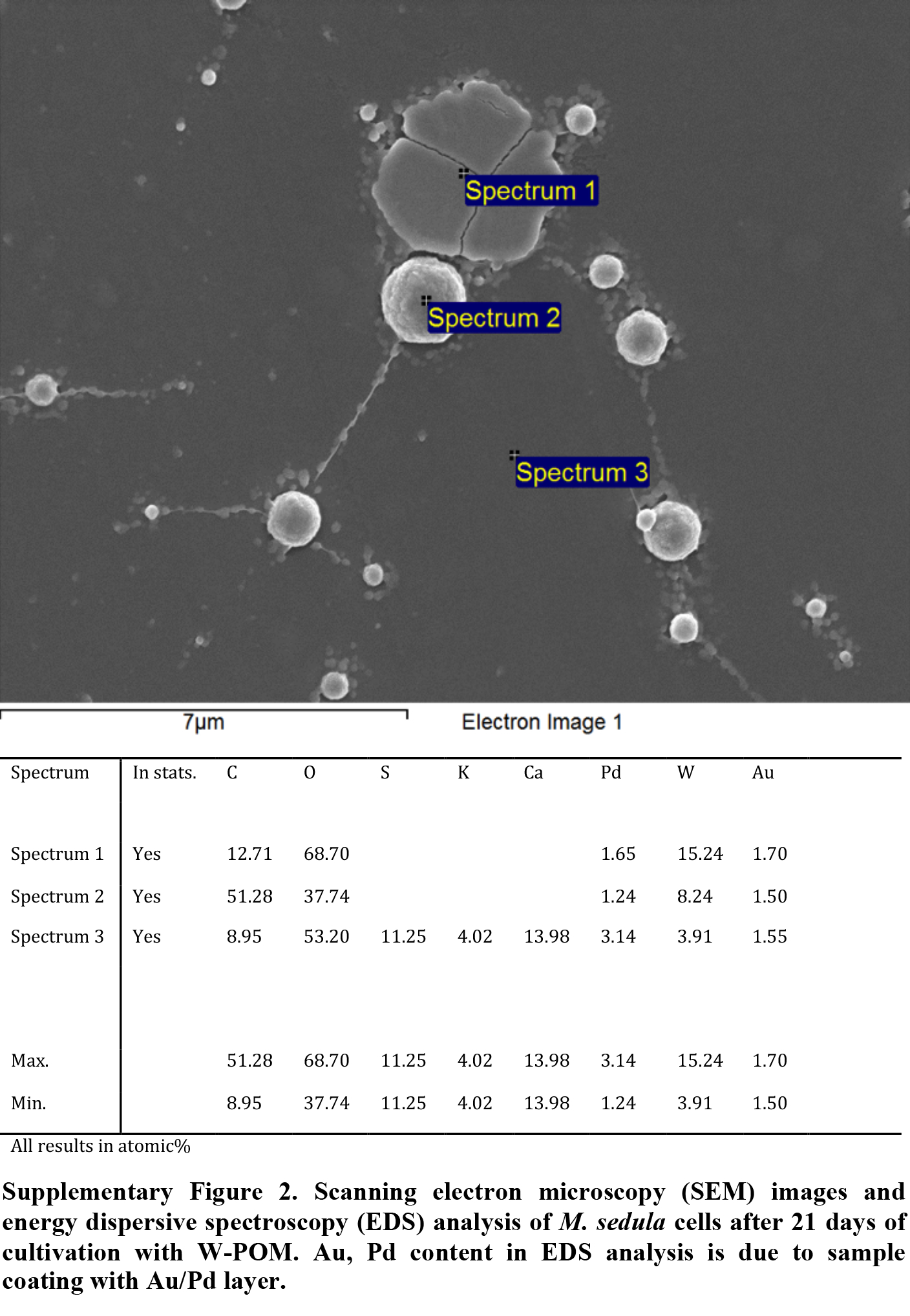

Supplement: Supplementary file 2 [file Image_2.JPEG]

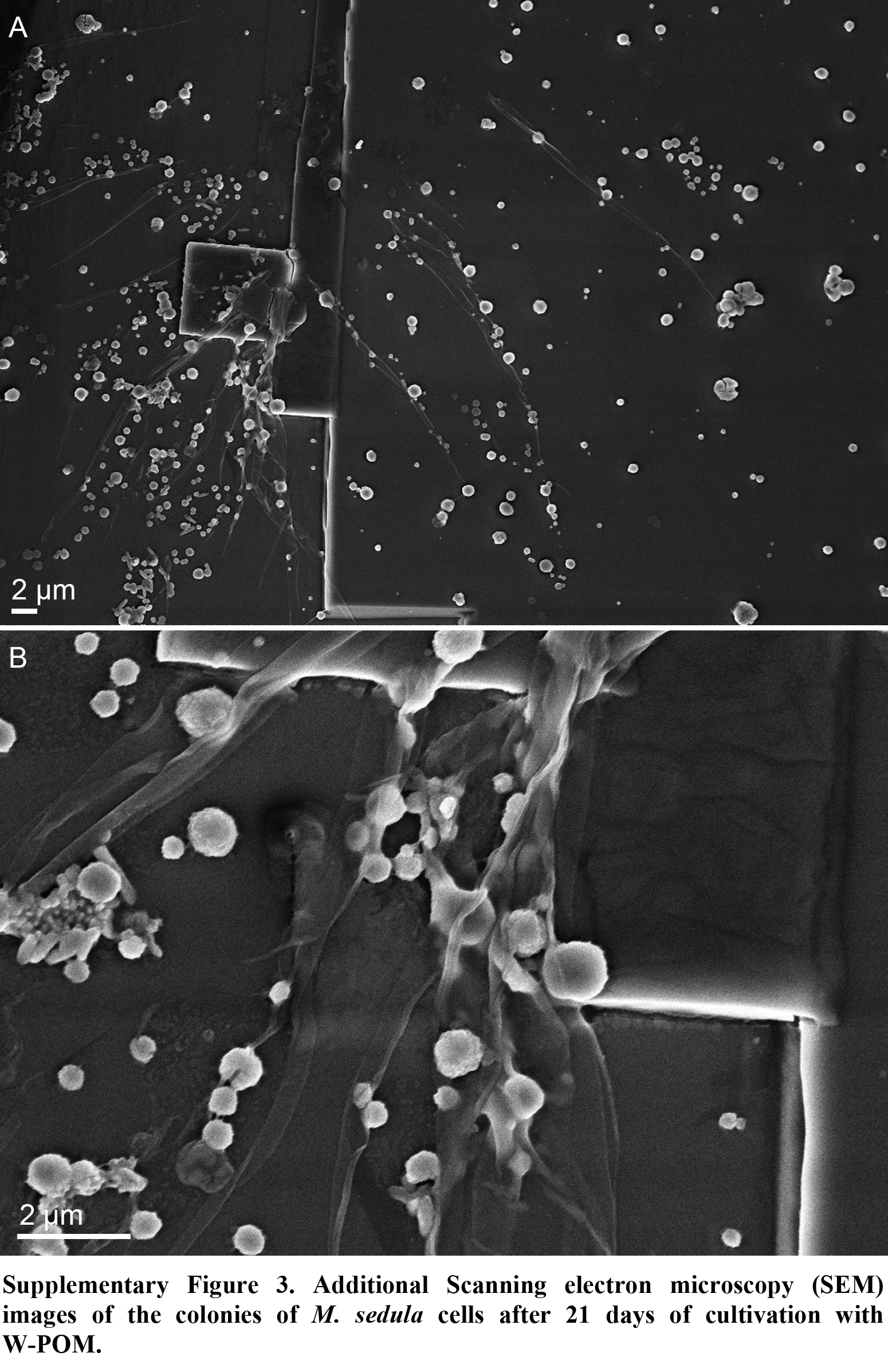

Supplement: Supplementary file 3 [file Image_3.JPEG]

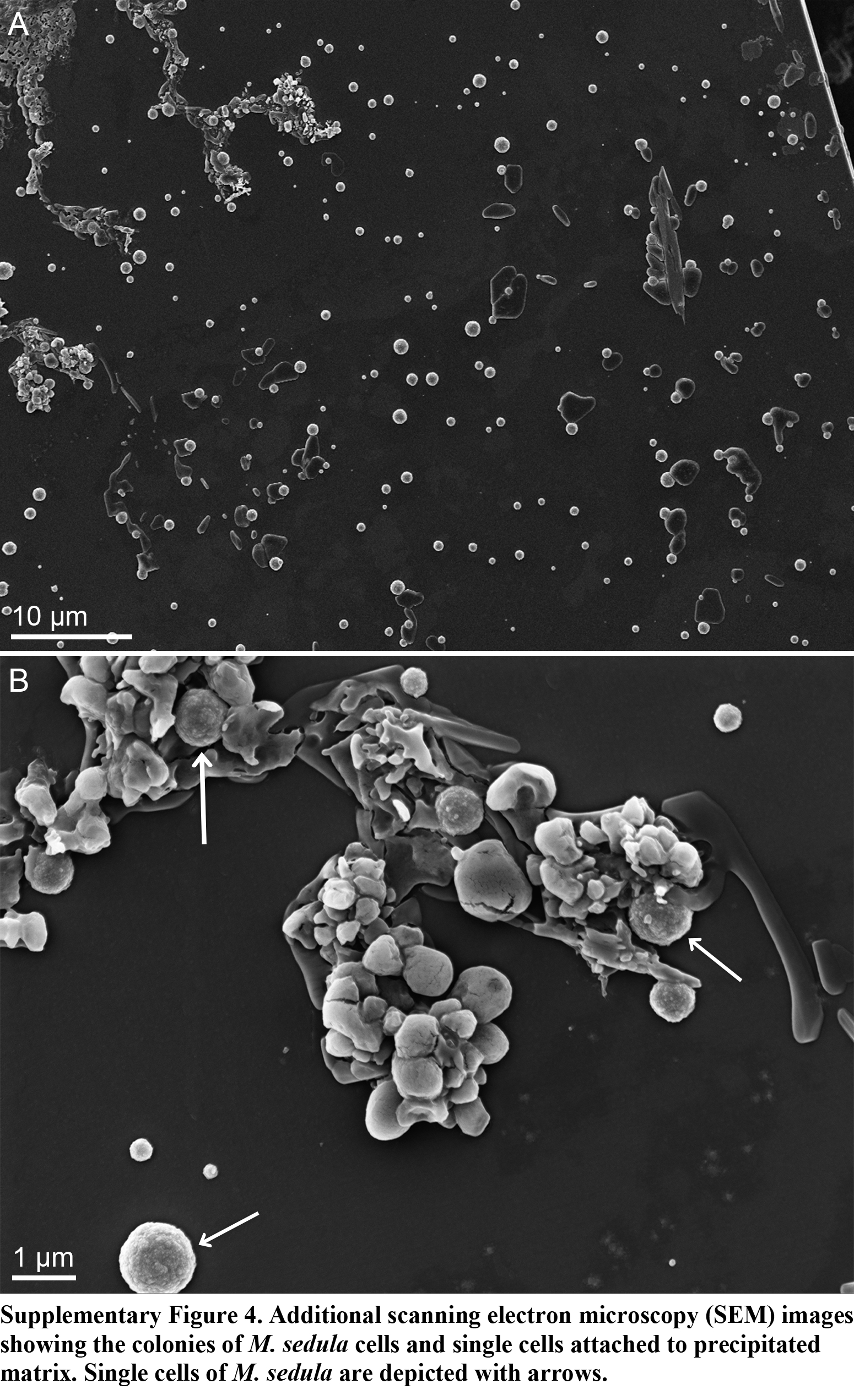

Supplement: Supplementary file 4 [file Image_4.JPEG]

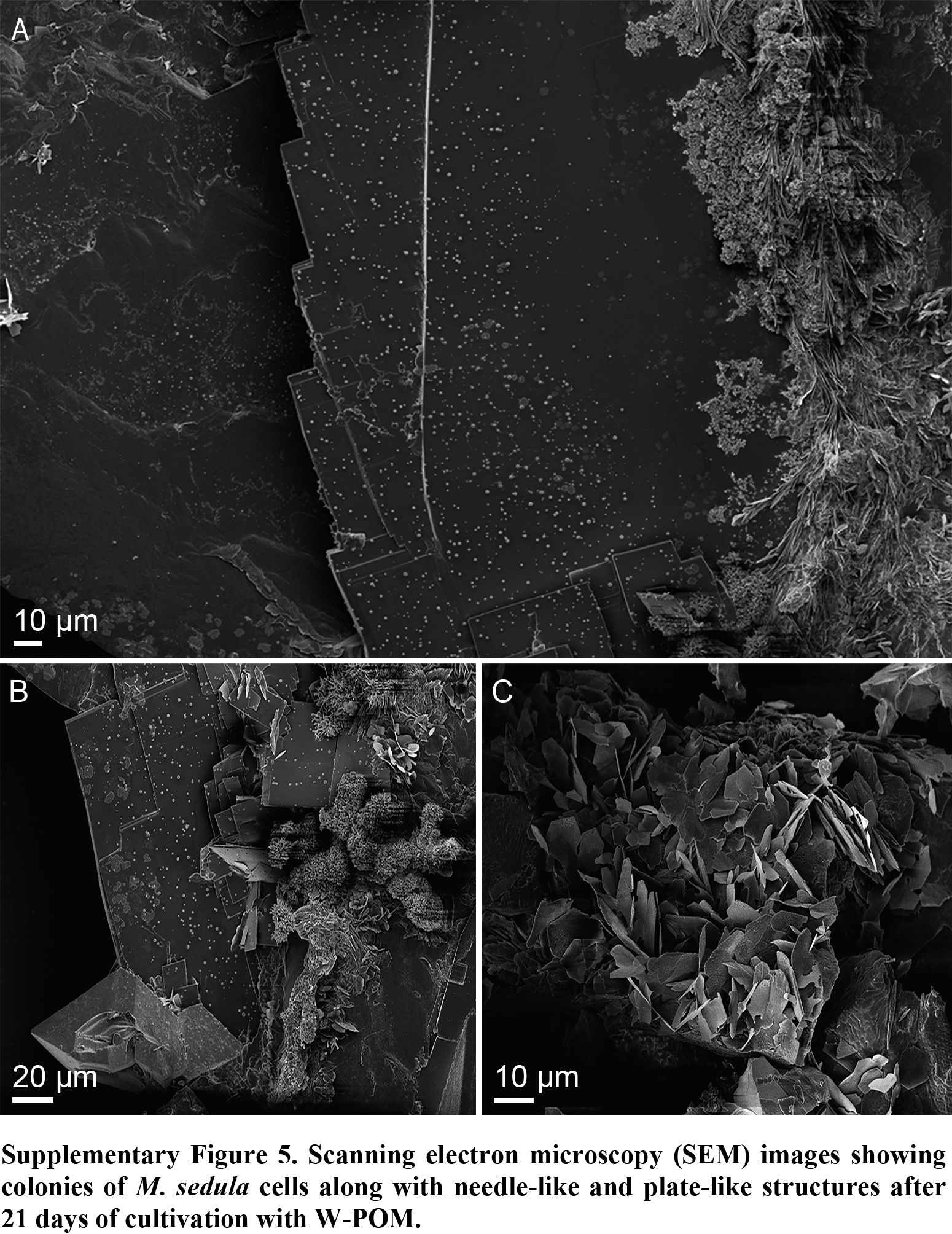

Supplement: Supplementary file 5 [file Image_5.JPEG]

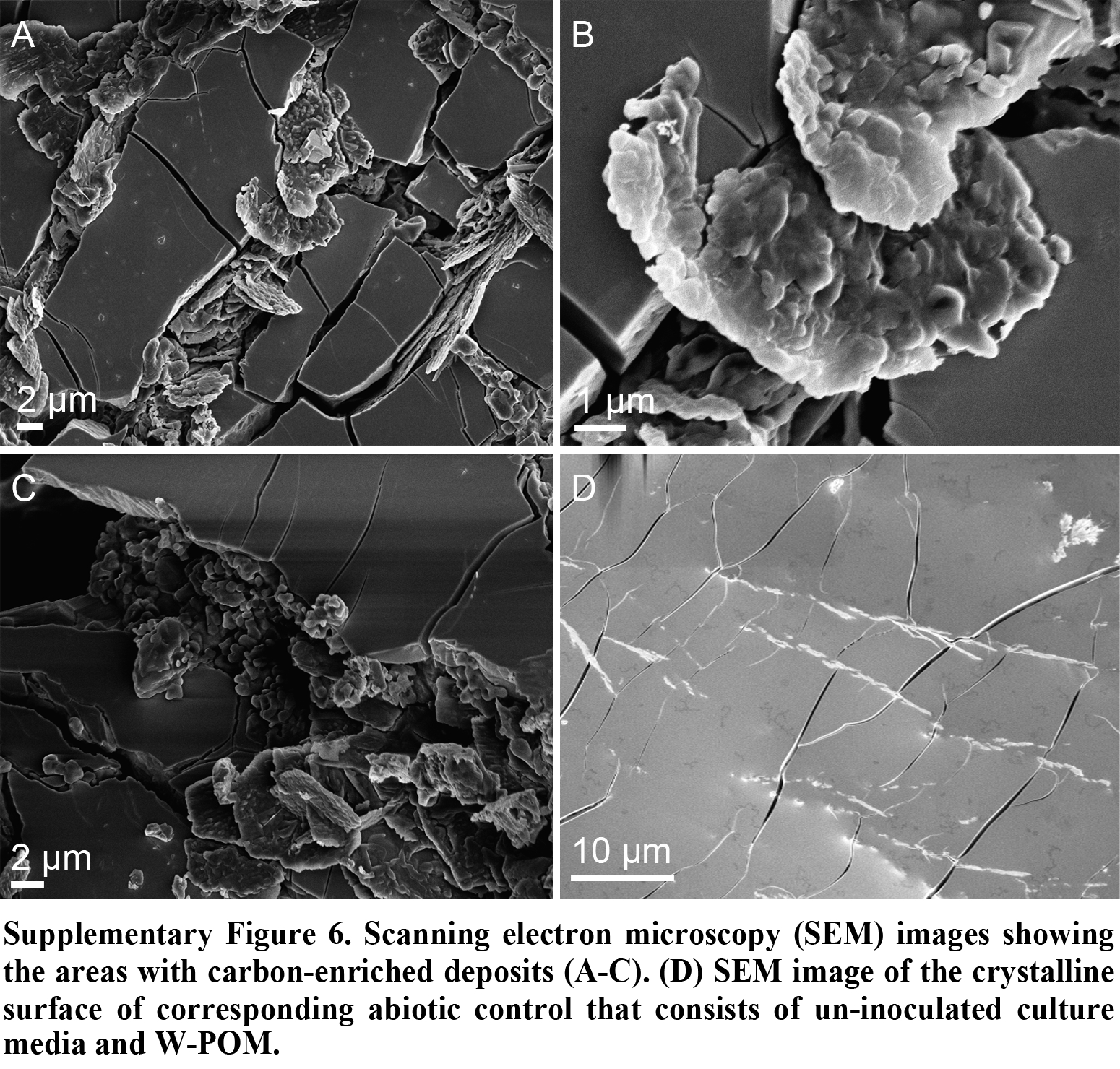

Supplement: Supplementary file 6 [file Image_6.JPEG]

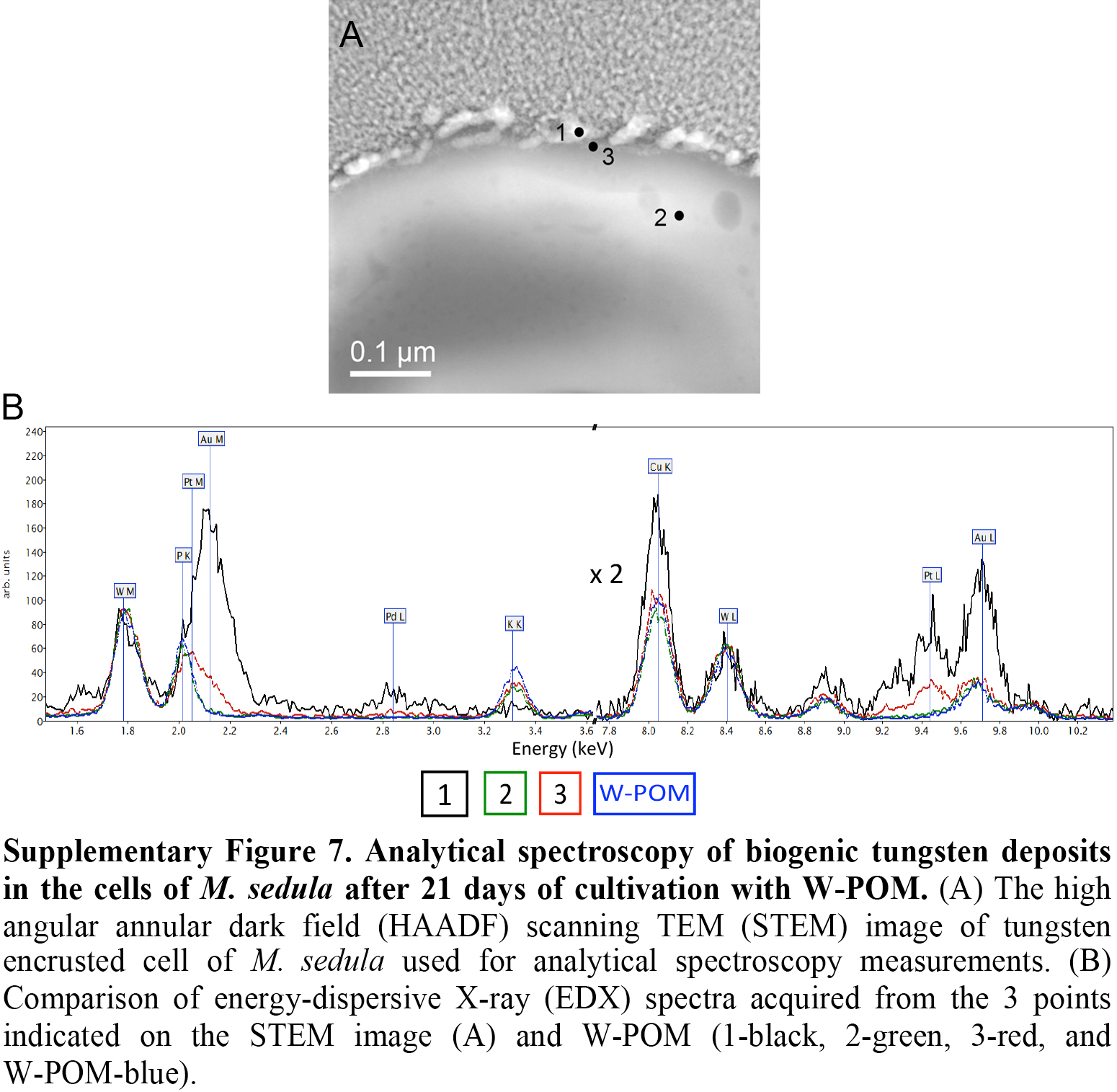

Supplement: Supplementary file 7 [file Image_7.JPEG]

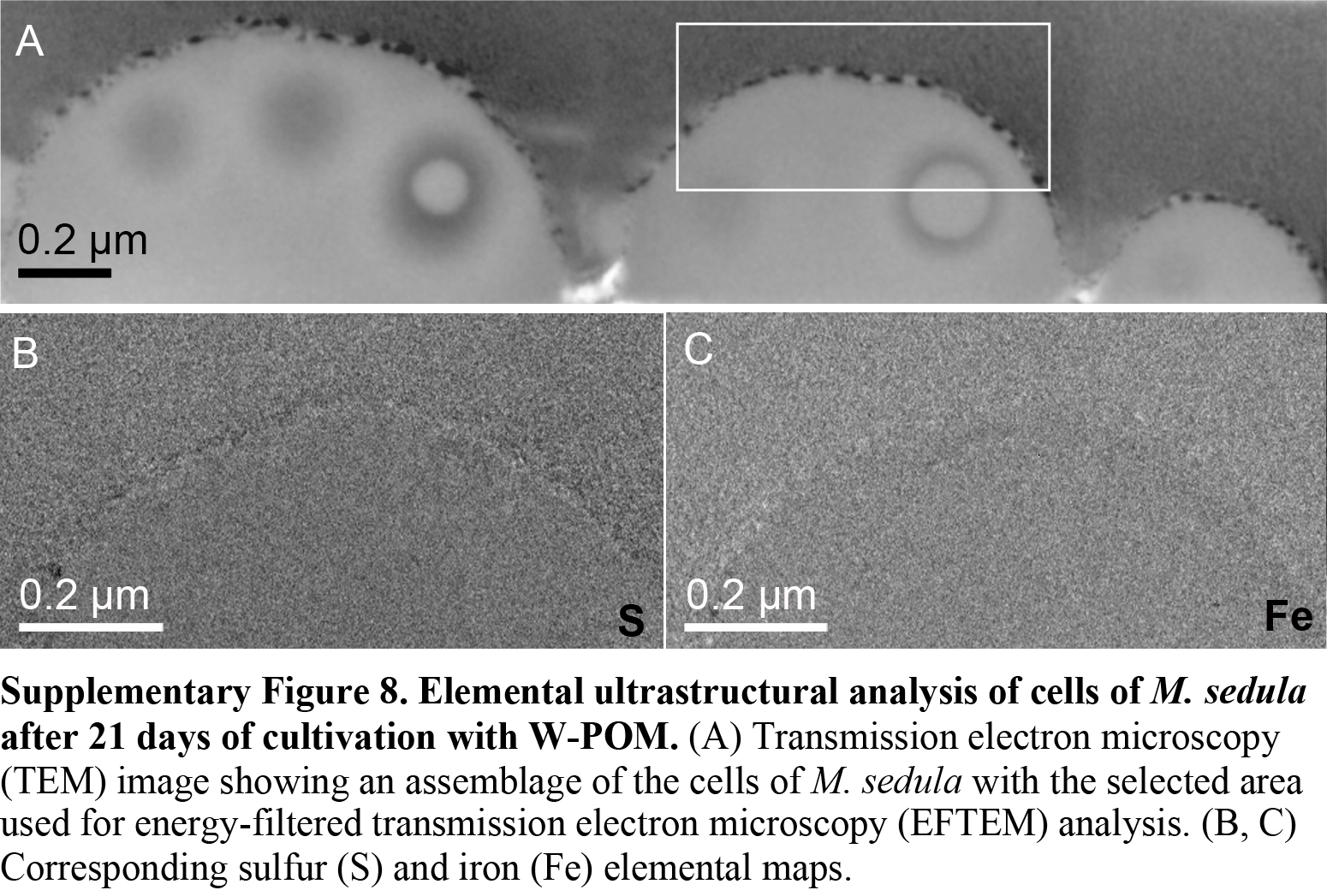

Supplement: Supplementary file 8 [file Image_8.JPEG]

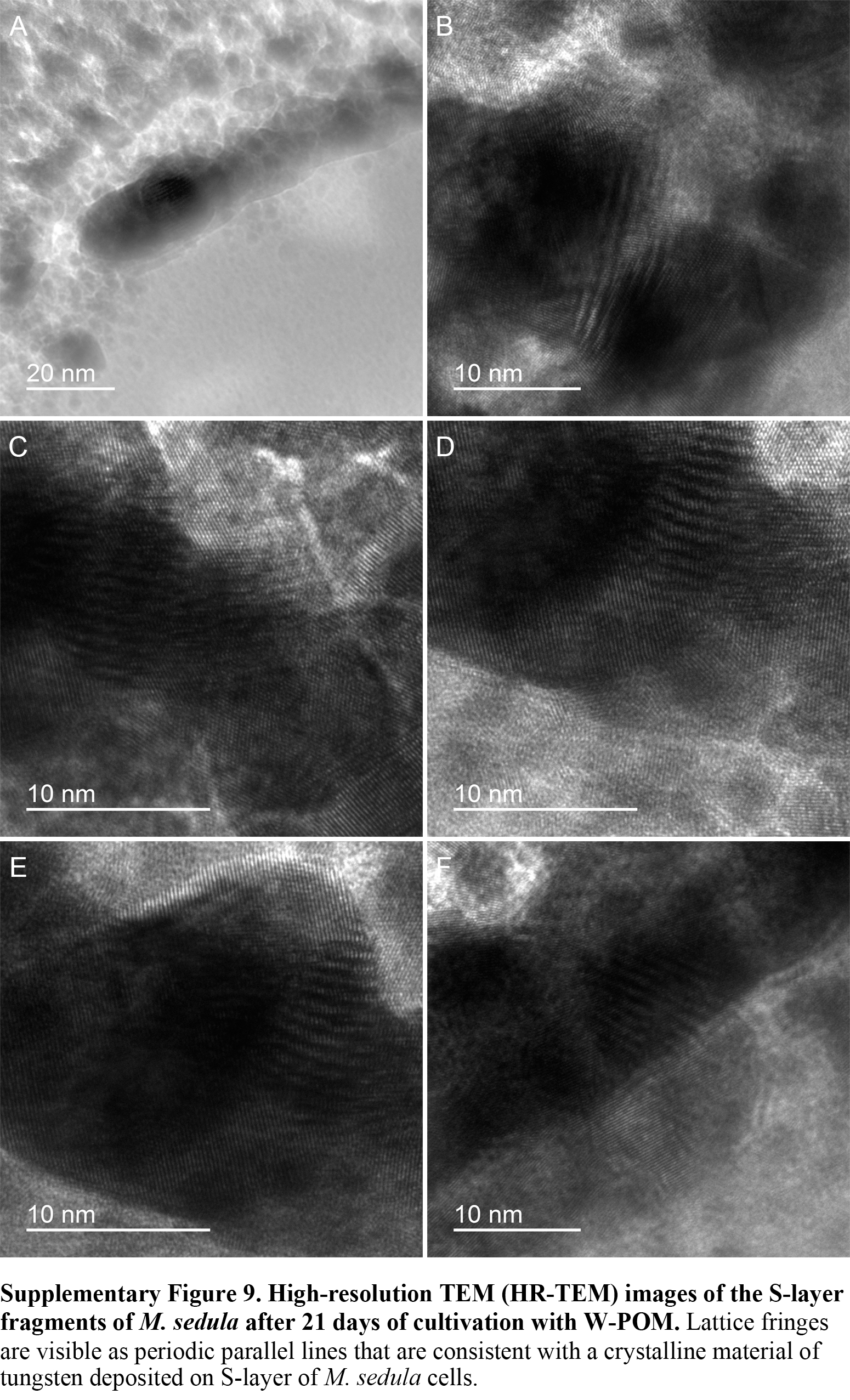

Supplement: Supplementary file 9 [file Image_9.JPEG]

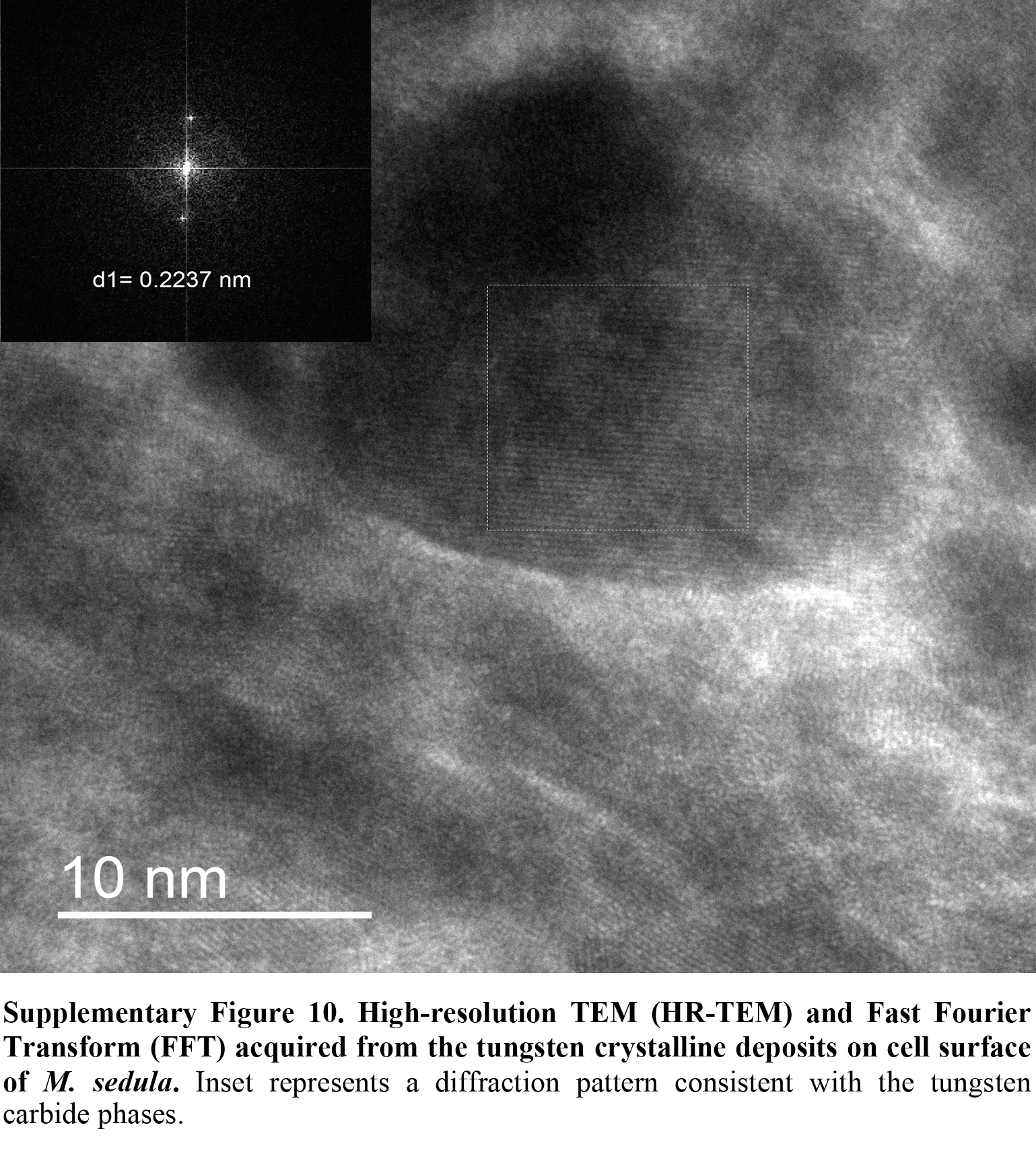

Supplement: Supplementary file 10 [file Image_10.JPEG]

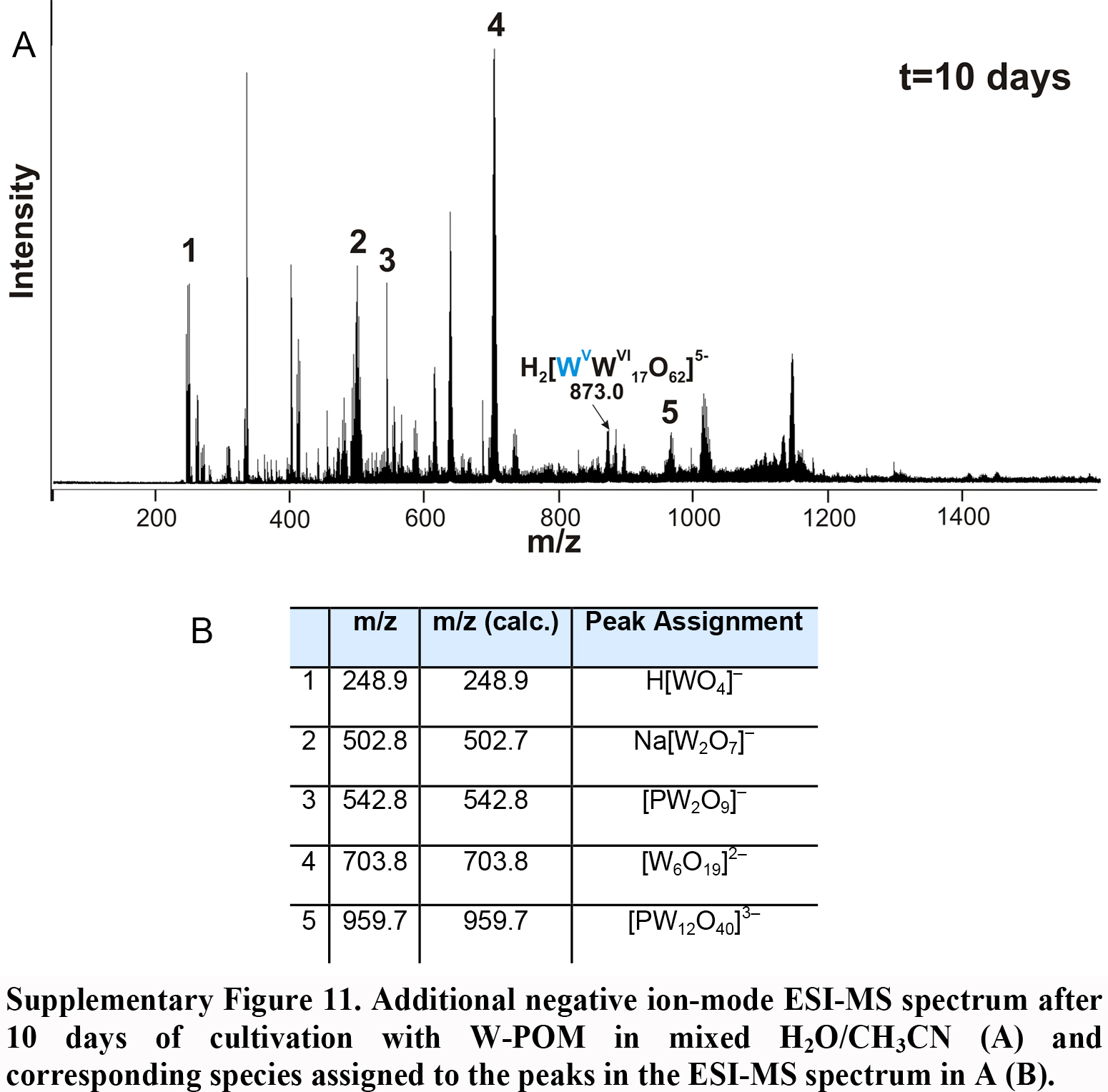

Supplement: Supplementary file 11 [file Image_11.JPEG]

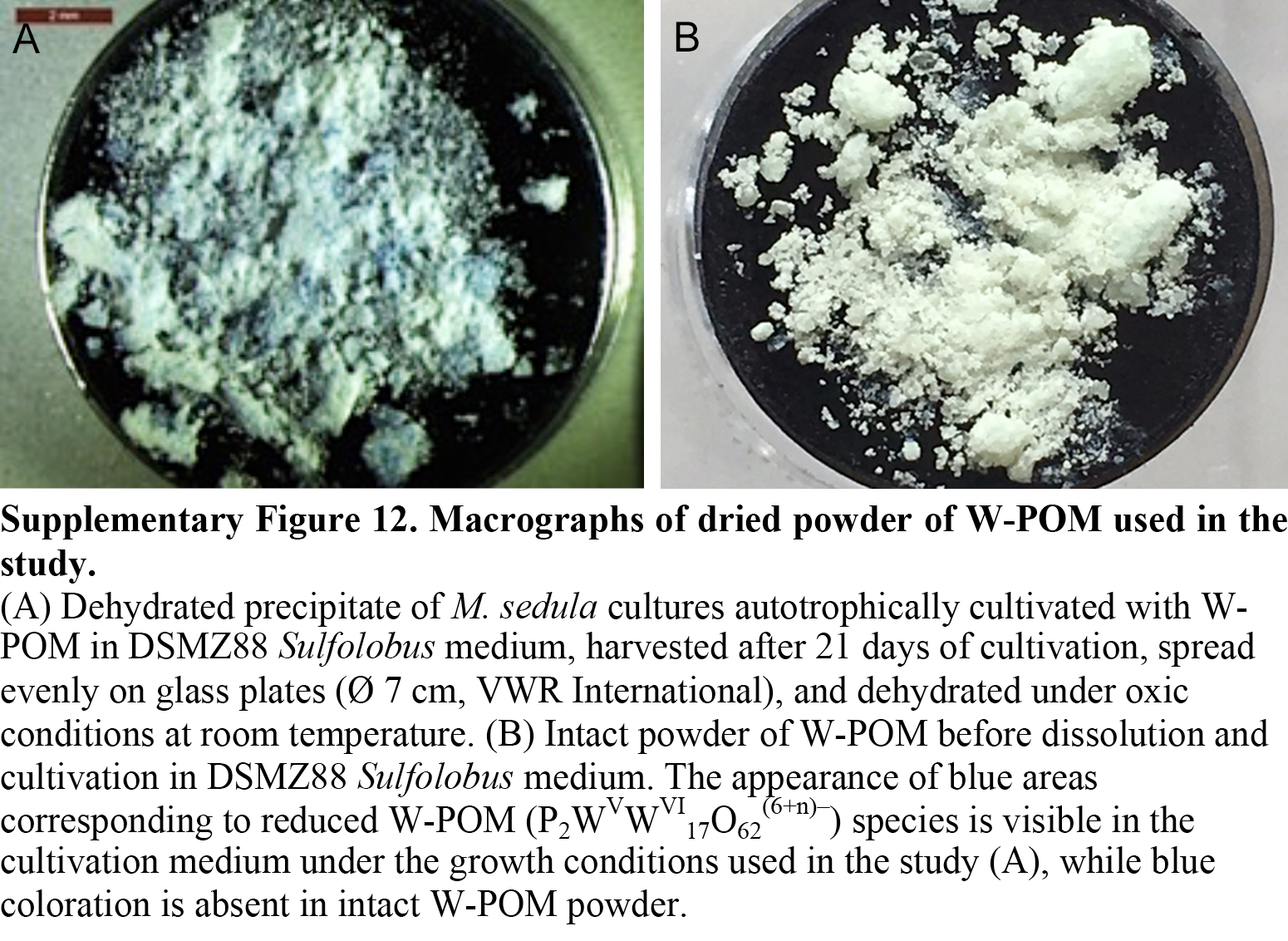

Supplement: Supplementary file 12 [file Image_12.JPEG]
