# Supplementary material for: Nanoscale Tungsten-Microbial Interface of the Metal Immobilizing Thermoacidophilic Archaeon Metallosphaera sedula Cultivated With Tungsten Polyoxometalate
Source: Front Microbiol. 2019 Jun 7;10:1267. doi: 10.3389/fmicb.2019.01267 (PMC6593293; doi:10.3389/fmicb.2019.01267)
Supplement: Supplementary file 13 [file Table_1.DOCX]

**Table S1. Average carbon, oxygen, and tungsten composition (atomic %) of the cells of *M. sedula* cultivated on W-POM**

|  | C | O | W |
| --- | --- | --- | --- |
| Mean % (+ standard deviation) *n =20* | 18.82±3.61 | 47.97±7.62 | 4.02±1.44 |
