# Supplementary material for: Nanoscale Tungsten-Microbial Interface of the Metal Immobilizing Thermoacidophilic Archaeon Metallosphaera sedula Cultivated With Tungsten Polyoxometalate
Source: Front Microbiol. 2019 Jun 7;10:1267. doi: 10.3389/fmicb.2019.01267 (PMC6593293; doi:10.3389/fmicb.2019.01267)
Supplement: Supplementary file 14 [file Table_2.DOCX]

**Table S2. Average element composition (atomic %) of the needles-like and plates-like structures precipitated from *M. sedula* cultures.**

|  | C | O | P | S | W |
| --- | --- | --- | --- | --- | --- |
| Mean % (+ standard deviation) *n =20* | 8.22 ± 7.21 | 43.45 ± 5.71 | 12.67 ± 12.47 | 15.04 ± 3.85 | 18.61 ± 3.85 |
