# Supplementary material for: Nanoscale Tungsten-Microbial Interface of the Metal Immobilizing Thermoacidophilic Archaeon Metallosphaera sedula Cultivated With Tungsten Polyoxometalate
Source: Front Microbiol. 2019 Jun 7;10:1267. doi: 10.3389/fmicb.2019.01267 (PMC6593293; doi:10.3389/fmicb.2019.01267)
Supplement: Supplementary file 15 [file Table_3.DOCX]

**Table S3. Average element composition (atomic %) of carbon-rich precipitates detected from *M. sedula* cultures.**

|  | C | O | W |
| --- | --- | --- | --- |
| Mean % (+ standard deviation) *n =20* | 70.08 ± 17.63 | 23.21 ± 14.71 | 3.93 ± 1.23 |
